# Dynamically Partitionable Autoassociative Networks as a Solution to the Neural Binding Problem

**DOI:** 10.3389/fncom.2012.00073

**Published:** 2012-09-28

**Authors:** Kenneth J. Hayworth

**Affiliations:** ^1^Janelia Farm Research Campus, Howard Hughes Medical InstituteAshburn, VA, USA

**Keywords:** binding problem, global workspace, ACT-R

## Abstract

An outstanding question in theoretical neuroscience is how the brain solves the neural binding problem. In vision, binding can be summarized as the ability to represent that certain properties belong to one object while other properties belong to a different object. I review the binding problem in visual and other domains, and review its simplest proposed solution – the anatomical binding hypothesis. This hypothesis has traditionally been rejected as a true solution because it seems to require a type of one-to-one wiring of neurons that would be impossible in a biological system (as opposed to an engineered system like a computer). I show that this requirement for one-to-one wiring can be loosened by carefully considering how the neural representation is actually put to use by the rest of the brain. This leads to a solution where a symbol is represented not as a particular pattern of neural activation but instead as a piece of a global stable attractor state. I introduce the Dynamically Partitionable AutoAssociative Network (DPAAN) as an implementation of this solution and show how DPANNs can be used in systems which perform perceptual binding and in systems that implement syntax-sensitive rules. Finally I show how the core parts of the cognitive architecture ACT-R can be neurally implemented using a DPAAN as ACT-R’s global workspace. Because the DPAAN solution to the binding problem requires only “flat” neural representations (as opposed to the phase encoded representation hypothesized in neural synchrony solutions) it is directly compatible with the most well developed neural models of learning, memory, and pattern recognition.

## Introduction

A gulf exists between our best cognitive science models of the mind and our best neuroscience models of the brain. Cognitive models hypothesize symbol assignments to variables, syntax-sensitive rule applications, and sequential, goal directed behaviors orchestrated by a central executive. Models incorporating these elements have demonstrated great success in modeling complex human behaviors including problem solving and language understanding. In contrast, neuroscience models hypothesize networks of neurons which function as pattern recognizers, associative memory stores, and feedback controllers. Models incorporating these elements have demonstrated that they can successfully model simple behaviors while simultaneously staying true to what anatomists and electrophysiologists have learned about biological neural networks.

It has proven very difficult to extend neuroscience models to encompass the more complex tasks which the cognitive models already easily handle. The difficulty lies in the “neural binding problem” – the act of assigning a symbol to a variable, and the act of applying a syntax-sensitive rule have no direct analog in current neuroscience models. I review this neural binding problem in detail below, showing why it really is a problem and why its most straightforward resolution (referred to here as the “anatomical binding hypothesis”) is unworkable in its most basic form.

Two main tactics have arisen over the years to overcome this difficulty (for reviews, see Roskies, 1999; Hummel, 2011). The first tactic has been an attempt to simplify cognitive models to eliminate symbol assignments and rules. The second tactic has been to posit significant additional complexity on top of the current neuroscience models (e.g., by positing neural synchrony and asynchrony among groups of neurons) which would allow them to handle symbol assignments and rules. To date, neither of these tactics has proved satisfactory.

This paper presents a third tactic. I show how symbol assignments and syntax-sensitive rules can be implemented using only traditionally accepted models of pattern recognition and associative memory (i.e., without the need for precise temporal synchrony). The solution presented is a novel variant on the anatomical binding hypothesis, but instead of associating each symbol with a particular pattern of neural firing (something which is not biologically plausible for reasons discussed below) I instead associate each symbol with a *piece* of a global stable attractor state. I introduce a new neural network formalism, the Dynamically Partitionable AutoAssociative Network (DPAAN), as an implementation of this solution. I then provide three examples of how a DPAAN can be used to solve the neural binding problem.

### Review of the binding problem

Cognitive models of brain function begin by assuming that the brain has some physical method for encoding symbols that represent features of the external world (Newell, 1990). Such symbols come in two types – atomic and composite. Atomic symbols form the basic vocabulary of a system and represent indivisible qualities like “blue,” “red,” “circle,” “square,” “above,” “below.” Composite symbols syntactically group atomic symbols into composite structures, for example “blue circle” or “the blue circle is above the red square.” Cognitive modelers have always assumed that the brain has mechanisms for encoding and manipulating such composite level symbols. That is, they have assumed that the brain has mechanisms for encoding syntax (the compositional arrangement of atomic symbols), recognizing syntactic differences (e.g., recognizing that “circle above square” differs from “square above circle”), and manipulating syntax. With this assumption cognitive modelers have been incredibly successful at modeling a wide range of human behaviors, and have produced general models meant to encompass the human cognitive architecture’s core features including skill learning, memory formation and retrieval, and goal directed behavior (Anderson et al., 2004).

There remains much debate as to how, and even whether, the brain’s biological neural circuits encode syntax. It is widely agreed that atomic-level symbols are encoded as unique patterns of activity over particular sets of neurons. For example, if a particular brain region is specialized for representing the color of an object, then each unique color will be associated with a particular pattern of activation across the neurons in this brain region. Let’s say there are five neurons in this color region and that we represent their joint activation with the vector **X**, then the particular activation pattern that represents the color red might be **X**^red^ = [10001] and the activation pattern that represents blue might be **X**^blue^ = [01100]. Similarly we may hypothesize another set of neurons **Y** specialized for representing shape, with activation patterns for representing “circle” and “square” as follows: **Y**^circle^ = [1100000], **Y**^square^ = [0000101]. To represent the composite idea “red circle” the system would simply set **X** = **X**^red^ and **Y** = **Y**^circle^, giving the joint activation across both sets of neurons as **X**; **Y** = [10001; 1100000].

However a difficulty arises when the system needs to represent a composite idea that utilizes the same modality (e.g., color) twice. There is no way to represent the idea “red circle and blue square.” Superimposing activation patterns for red and blue give a pattern that is neither (i.e., **X** = **X**^red^ + **X**^blue^ = [11101]), likewise for the shape modality. (**Y** = **Y**^circle^ + **Y**^square^ = [1100101]). And even if this “superposition catastrophe” (von der Malsburg, 1999) could be worked around, there is still nothing in the resulting joint representation (**X**; **Y** = [11101; 1100101]) that distinguishes it from “blue circle and red square.” The root of the problem is that the above representational scheme has no way to signal that the color red is “bound” with the shape circle and that the color blue is bound with the shape square. This is the classic binding problem of how to represent two visual objects simultaneously; however the binding problem is in no way limited to visual representation. The same issues arise in representing non-visual concepts like “John loves Mary” vs. “Mary loves John.” To properly represent the concept “John loves Mary” one needs some way to bind the atomic symbol “John” to the “subject” slot of the sentence and to bind the atomic symbol “Mary” to the “object” slot. If a single set of neurons is used to represent all persons then again one runs into the same superposition and ambiguity problems.

Perhaps the simplest proposed solution to this binding problem is to posit two independent sets of neurons for each modality. For example, instead of having just two sets of neurons one could instead have four sets of neurons (**X**_1_ to represent the color of object #1, **Y_1_** to represent the shape of object #1, **X**_2_ to represent the color of object #2, **Y_2_** to represent the shape of object #2). Now if one wanted to represent the concept “red circle and blue square” one would produce the joint activation **X**_1_; **Y_1_**; **X_2_**; **Y_2_** = [10001; 1100000; 01100; 0000101]. This appears to resolve all of the problems discussed above, and indeed it does since this is exactly how a computer programmer would solve such a problem. The programmer would simply assign a variable (**X_1_**) for the color of object #1 and a separate variable (**X_2_**) for the color of object #2, and two variables **Y_1_**, **Y_2_**) for the objects’ shapes. At the electrical implementation level such variables exist each as a separate 32-bit memory register (each register composed of a string of 32 separate single bit storage cells) in the computer’s CPU. The analogy is that each neuron is like one of these single bit storage cells. Any program using these four variables is written to understand that variables **X_1_** and **Y_1_** are referring to the same object and variables **X_2_** and **Y_2_** are also referring to a single object distinct from the first object.

This solution, which I will refer to as the “anatomical binding hypothesis^1^,” may work for computers but it has long been rejected by theoretical neuroscientists as a model for how the brain works. In the section “Arguments Against the Anatomical Binding Hypothesis” below I will carefully lay out the arguments that have been made against this anatomical binding hypothesis showing that they do indeed rule out simplistic neural implementations. However the body of this paper will show that these arguments against the brain’s use of anatomical binding can be overcome with a particular type of neural implementation (the DPAAN) and training regime.

Before making these arguments however I will need to first briefly review the standard theory for how a single object is encoded by the visual system and show how this theory can be straightforwardly extended to encode multiple objects simultaneously using anatomical binding. This will set the stage for a discussion of anatomical binding’s key problem as well as my proposed solution to this problem.

### The standard model of visual object representation

Before we sketch out a model of the visual system which uses anatomical binding to encode two objects we need to first have a firm grasp on our current best model of how the visual system is thought to encode a *single* object. In this standard theory, neural fields like **X_1_** and **Y_1_** are activated by a “feature hierarchy” of neurons whose receptive fields cover the entire visual field (Felleman and Van Essen, 1991; Kobatake and Tanaka, 1994). It is now well known how to create such a visual feature hierarchy. Such networks have a long history in visual neuroscience starting with the Neocognitron model of Fukushima (1980) which was a computational implementation and extrapolation of the simple and complex visual cortex cell models proposed by Hubel and Wiesel. Other feature hierarchy models include VisNet (Rolls and Stringer, 2006), HMAX (Riesenhuber and Poggio, 1999), SEEMORE (Mel, 1997), and Chorus of Fragments (Edelman and Intrator, 2000). In fact, this neural formalism is so prevalent in visual neuroscience, and so well supported by experimental and anatomical data, that its key elements have been called “The Standard Model” (Riesenhuber and Poggio, 2003) of visual cortex function.

Such feature hierarchy models of the visual system provide a neural implementation-level explanation as to how the retinal image of an object at an arbitrary position in the visual field can be transformed into a set of translation invariant “symbols” describing the object. Given the extensive literature already in existence on such feature hierarchy models, I will provide only a brief overview of such operation here.

In a feature hierarchy model, the retinal image is first locally processed by a set of simple feature detecting cells each of which looks for a particular target visual feature like a contrast or color-defined edge boundary at a particular orientation. Such cells are analogous to the V1 simple cells found by Hubel and Wiesel. Collections of such simple cells which look for the same target feature but whose receptive field locations are translated slightly relative to each other are then fed onto another type of cell – a complex cell-one layer higher in the feature hierarchy. These complex cells respond if any of their inputs are active, thus providing a positive signal if the target feature is present anywhere within their larger receptive field. As suggested first by Fukushima (1980) this pattern of simple (S) cells and complex (C) cells is repeated across several levels, wherein each new S-cell layer responds to more visually complicated target features (e.g., sharp corners) and each new C-cell layer contains cells whose receptive fields cover a larger fraction of the visual field. In the limit there is a final C-cell layer whose cells respond if their target feature is present anywhere within the retinal image, and whose feature vocabulary is such that every visual object which we can recognize will generate a distinct and unique pattern of activation over this final C-cell layer (Serre et al., 2005). Some cells within this final C-cell layer will respond only to the shape characteristics of the viewed object, and if we group those cells together they correspond precisely to the **Y_1_** shape neurons described above. Similarly, some cells within this final C-layer will respond only to the color (and surface texture) characteristics of the viewed object, and if we group those cells together they correspond precisely to the **X_1_** color neurons described above. Anatomically separated cortical regions have been identified in the primate brain whose responses roughly correspond to such shape and color regions (Kandel and Wurtz, 2000).

Such a model breaks down if there is more than one object in the visual field and if there are significant background features. The ready solution is to include a spotlight-like visual attention mechanism into the model wherein the features of only one part of the retinal image are allowed to drive higher levels of the feature hierarchy. Many neural models of such an attention mechanism have been described in the literature and there is extensive experimental evidence that such an attentional mechanism is built into the primate cortex’s visual feature hierarchy (e.g., Reynolds and Desimone, 1999). Such an attentional mechanism not only solves the problem of how to ensure that the activation patterns in the final **X_1_** and **Y_1_** neural fields provide clear shape and color symbols (avoiding the superposition catastrophe of corruption by overlapping symbols), but it also ensures that **X_1_** and **Y_1_** are always encoding the color and shape of the same visual object thus avoiding illusory conjunctions (Treisman, 1999). Finally, the inclusion of this attentional mechanism provides a straightforward way for the brain to encode the retinal position of the attended object separately from the object’s identity. This is done by a third field of neurons (let’s call it **Z_1_**) which encodes the retinal position of the spotlight of attention itself. Thus the triplet of neural fields (**X_1_**, **Y_1_**, and **Z_1_**) would encode the color, shape, and position respectively of a single visual object.

### Extending the standard model to simultaneously represent two objects by anatomical binding

The above “Standard Model” of the visual system is an oversimplification of the brain’s actual functioning but it conveys the general consensus model which has been gleaned from hundreds of experiments over the years (Riesenhuber and Poggio, 2003). For our purposes here its main structure will be assumed to be correct. It is important to understand that there is no “binding problem” in the above description of how the visual system represents a *single* object. This model offers a perfectly reasonable explanation for how the color of an object is “bound” to its shape (e.g., Treisman, 1999), and for how an object can be recognized in a translation invariant manner while still having its position information “bound” to it. Since the attentional spotlight locks on to only one object at a time, the triplet of neural fields (**X_1_**, **Y_1_**, and **Z_1_**) are guaranteed to describe different aspects (color, shape, and position) of the same object.

The true “binding problem” enters when one asks how such a system could represent two (or more) visual objects simultaneously – something which psychophysical experiments have demonstrated humans readily achieve (e.g., Kawahara and Yamada, 2006). Several different theories have been put forward in the literature for how the Standard Model could be modified to allow such simultaneous representation of two objects. (Hayworth et al., 2011) reviews these different proposals in depth. For a variety of reasons covered in that paper the most straightforward modification seems to be to posit multiple spotlights of attention which can be independently trained on the separate objects. Although visual attention is usually described as a single “spotlight,” there is extensive evidence that the human visual system in fact supports multiple simultaneous spotlights of attention (McMains and Somers, 2004; Cavanagh and Alvarez, 2005; Kawahara and Yamada, 2006; Yamada and Kawahara, 2007; Hayworth, 2009) and including this known fact into the Standard Model picture seems a straightforward route to allowing the simultaneous encoding of multiple objects^2^.

**Figure 1:**
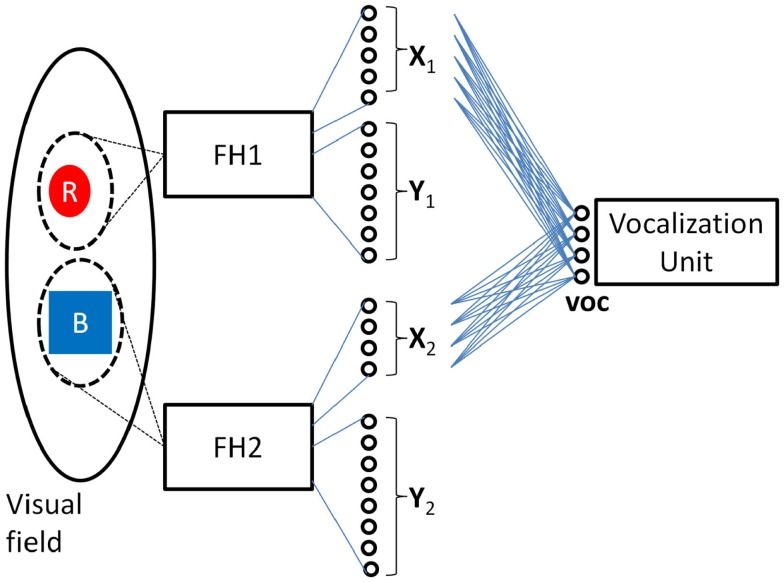
**Model of visual perception consisting of two feature hierarchies (boxes labeled FH1 and FH2) each with their own spotlight of attention**. The final output layer of FH1 consists of the neural fields **X_1_** and **Y_1_** which encode the color and shape of the object within FH1’s spotlight of attention in a translation invariant manner. Similarly, the final output layer of FH2 consists of the neural fields **X_2_** and **Y_2_** which encode the color and shape of the object within FH2’s spotlight of attention. This model of the visual system is a straightforward extension of the Standard Model which uses multiple spotlights and anatomical binding to encode two objects simultaneously.

Figure 1 depicts the key features of such a system in which two independent feature hierarchies (labeled FH1 and FH2) are each associated with their own spotlight of attention which I will refer to as spotlight #1 and #2 respectively. FH1’s final C-cell layer is composed of the neural fields **X_1_** and **Y_1_** which encode the color and shape respectively of the object highlighted by spotlight #1, and FH2’s final C-cell layer is composed of the neural fields **X_2_** and **Y_2_** which encode the color and shape respectively of the object highlighted by spotlight #2^3^. The role of the vocalization unit depicted will be discussed later.

This particular model of the visual system, termed the Multiple Slots Multiple Spotlights model, is explored in more detail in Hayworth (2009) and some experimental evidence in its favor is presented in Hayworth et al. (2011). This extension of the Standard Model to handle the representing of two objects simultaneously is a concrete example of the anatomical binding hypothesis described above. Even though this model seems to be capable of solving the task of representing two objects simultaneously most theoretical neuroscientists would immediately dismiss it (and similar models using anatomical binding) on the grounds that it is not biologically plausible. In the next section I carefully lay out the arguments that have been made against this anatomical binding hypothesis showing that they do indeed rule out simplistic neural implementations.

### Arguments against the anatomical binding hypothesis

The central argument put forward against the anatomical binding hypothesis is as follows: The solution seems to require that there be a one-to-one correspondence between the neurons in **X_1_** and the neurons in **X_2_** in precisely the same way there is a one-to-one correspondence between each bit of two 32-bit computer registers. A human-designed computer is literally wired up to ensure such a one-to-one correspondence between the bits of each memory register, and all the computations performed on a computer (e.g., comparing two registers, adding the contents of two registers and transferring the sum to a third, etc.) are only possible because of this one-to-one correspondence between bits. Since a biological brain is wired up by developmental processes, it is not credible to expect such a one-to-one correspondence between particular neurons.

This argument is usually put in slightly different terms: If the brain did represent the colors and shapes of two objects by using separate sets of neurons (**X_1_**; **Y_1_)** and (**X_2_**; **Y_2_)**, then the pattern representing “red” on **X_1_** would have nothing in common with the pattern representing “red” on **X_2_**, thus thwarting intelligent actions which require such understanding.

In the example model of the visual system presented in the section above, the **X_1_** neurons represent the last layer of the FH1 feature hierarchy whose neural connectivity was presumably produced through self-organized learning (e.g., Foldiak, 1991; Wallis et al., 1993). Similarly the **X_2_** neurons represent the last layer of the FH2 hierarchy. Given the vagaries of development it is unlikely that these two separate sets of neurons would even have the same number of cells. There is certainly no chance that one would find a one-to-one correspondence between individual cells in each area such that for all colors corresponding cells would always have the same activation. What this means is that if ${\text{X}}_{1}^{\text{red}}$ = [10001] then ${\text{X}}_{2}^{\text{red}}$ could be just about any other pattern and there is no way to line up the neurons between ${\text{X}}_{1}^{\text{red}}$ and ${\text{X}}_{2}^{red}$ and compare them. What is taken for granted in a human-designed computer cannot be taken for granted in the self-wired brain.

More importantly, if the brain did represent the colors of two objects by using two separate sets of neurons then any neural associations that were learned for one set would not “transfer” to the other. For example, suppose such a system encoded that the object highlighted by spotlight #1 (call this object #1) was colored red by activating the pattern [10001] on **X_1_** neurons, and suppose that there is another set of neurons **voc** whose job is to deliver specific motor commands to a vocalization unit (see Figure 1). Supervised training (e.g., by mimicking a parent’s voice when viewing a red object as object #1) could result in a set of synaptic connections between **X_1_** and **voc** that associate the **X_1_** pattern for red with the **voc** pattern needed to pronounce the word “red” out loud. Similar training would create the synapses between **X_1_** and **voc** allowing the system to vocalize the word “blue” when **X_1_** registers [01100]. The key question is this: “Would the training that allows the system to pronounce the color of object #1 transfer such that the system could also pronounce the color of the object highlighted by spotlight #2?” The answer is no. Training would modify synapses between **X_1_** and **voc** only. Since, by hypothesis, **X_2_** neurons were not even active during this training (and since the pattern for “red” on **X_2_** is different than the pattern for “red” on **X_1_**) there is no way that the appropriate synapses could have been modified to create the proper associations between **X_2_** and **voc**. Neuroscientists believe that these types of learned associations (via modifications of synaptic connection between two sets of neurons) are the basis of all computation in the brain. These associations are the *only* thing that gives a pattern of activation meaning to the system, and since associations learned for **X_1_** do not transfer to **X_2_**, the system has no way of understanding if **X_1_** and **X_2_** activation patterns are representing the same or different colors.

A summary of the above argument is that using two independent sets of neurons to represent two objects simultaneously does not work because it violates “role-filler independence” (Hummel et al., 2004; Hummel, 2011). There are two “roles” in this case: the color of object #1 (represented by **X_1_** neurons) and the color of object #2 (highlighted by spotlight #2 and represented by **X_2_** neurons). We would like to fill either of these roles with the symbol “red” and have its meaning remain constant across these roles. However if we have built up meaning-embedded associations only with the **X_1_** neurons then the symbol’s meaning will not be constant across the two role slots.

Arguments like these have convinced many theoretical neuroscientists that the way the brain solves the binding problem cannot be to simply use multiple independent sets of neurons to represent different objects (or sentence roles, etc.). Instead it is often assumed that there must be only one set of neurons that represents each individual modality. Representation of multiple objects then requires a form of time-sharing among the different objects. This is the “binding by neural synchrony” hypothesis (Hummel and Biederman, 1992; von der Malsburg, 1995). According to that hypothesis, to represent “red circle and blue square” only one set (**X**) of color cells would be used, and only one set (**Y**) of shape cells, but these groups of cells would alternate back and forth between representing “red circle” (**X**; **Y** = [10001; 1100000]) and representing “blue square” (**X**;**Y** = [01100; 0000101]). Much theoretical, modeling, and experimental work has been devoted to exploring this binding by neural synchrony hypothesis with mixed results (Roskies, 1999; Shadlen and Movshon, 1999). What is certain, however, is that it has proved much more difficult to develop theories for encoding, manipulating, and storing such “phase encoded” activations than it has to develop theories of how to encode, manipulate, and store “flat” activation patterns resembling the vector strings of ones and zeros above. For example, networks of simple perceptrons offer a straight forward explanation for the encoding of such flat activation vectors, and Hebbian-style weight modification offers an explanation for how such flat activation vectors could be stored as stable attractors in associative networks allowing for simple models of content addressable recall.

In this paper I will show that the binding problem can be solved in a biologically plausible manner using only flat neural vector representations, with no need to assume precise temporal synchrony between sets of neurons. As such, the wealth of models for how flat activation vectors can be encoded and stored in memory will be immediately applicable.

### Outline of the DPAAN solution

Using the visual model depicted in Figure 1 as a concrete example, let us crystallize the above arguments against anatomical binding and derive requirements that any such scheme must meet to avoid these pitfalls. To review, the box FH1 represents a hierarchy of feature extracting cells which respond to features across the entire visual field but whose responses are such that they only respond to the object locked onto by attentional spotlight #1. FH1’s final layer of cells consists of the neural fields **X_1_** and **Y_1_** which represent the extracted color and shape respectively of the object (#1) locked onto by spotlight #1. The box FH2 represents a separate feature hierarchy whose final layer consists of **X_2_** and **Y_2_** representing the extracted color and shape of the object (#2) locked onto by spotlight #2. Lastly there is a trainable association network connecting the neural field **X_1_** with the field **voc**, and we assume that this network has been trained to correctly vocalize the color of the object highlighted by spotlight #1 which is represented on the **X_1_** neurons. A separate association network connecting the neural field **X_2_** with **voc** is assumed to have *not* been so trained.

Now the two central arguments against this anatomical binding scheme can be put as follows:

1. How could the larger neural system using this anatomical binding scheme determine whether object #1 and object #2 had the same or different colors (or shapes, etc.)?
2. How could the system vocalize the color of the object represented by **X_2_** by making use of the associations learned on a different set of neurons (e.g., the **X_1_**-to-**voc** connections)?

These will be the basic requirements we must satisfy. I will later show how satisfying these requirements can lead to a general purpose system that has all the syntactic processing capabilities assumed by the most advanced cognitive models.

First we should realize that if the brain was designed like a computer the answers to these questions would be very straightforward. A human designer would ensure that the code for the colors used by **X_1_**, **X_2_**, and even **voc** would all be identical. Thus determining if object #1 and object #2 had the same or different colors would simply require comparing corresponding neurons in **X_1_** and **X_2_**; and interfacing with the Vocalization Unit’s abilities would merely involve transferring the contents of either **X_1_** or **X_2_** directly to **voc**. In such a human-designed system, FH1, FH2, and the Vocalization Unit would still have to be trained to perform their individual tasks but such training would be *localized* within these individual modules.

Because the brain has no top-town designer we cannot assume that FH1, FH2, and the Vocalization Unit use the same pattern code for corresponding color symbols. Because of this (and the roll-filler independence argument) “translations” between codes must be performed via association networks (like the **X_1_**-to-**voc** connections). When only two modules (like FH1 and the Vocalization Unit) must communicate, such translation (via a trained association network) presents no problems; in fact, such translation is traditionally simply thought of as part of the training needed within the Vocalization Unit’s network (or FH1’s). One recognizes a problem only when many modules must communicate with each other like in the visual binding problem above.

Invoking a “world languages” analogy, we can think of **X_1_** (i.e., FH1) as speaking a different “language” of neural patterns then **voc** (i.e., the Vocalization Unit). In this analogy, the purpose of the trainable neural connections between **X_1_** and **voc** is to *translate* one language into the other. Both the perceptual field **X_1_** and the motor field **voc** have internal symbols for the concept “red” but **X_1_** speaks, by analogy, Spanish (i.e., “rojo”) and **voc** speaks French (i.e., “rouge”)^4^. Since they do not speak the same language natively one must be trained to understand the other’s language. Training the **X_1_**-to-**voc** synaptic connections can be analogized as **voc** learning to translate **X_1_**’s language (i.e., Spanish) into its own (i.e., French). In this “world languages” analogy **X_2_** (i.e., FH2) also has the semantic concept “red” but speaks yet a third language (say German in which “red” is pronounced “rot”). To allow the Vocalization Unit to vocalize what color is represented by the **X_2_** neurons the **X_2_**-to-**voc** connections must be trained as well so that **voc** can translate **X_2_**’s specific neural code into its own. In operation, when the spotlights are allowed to lock onto separate objects, the larger system would have to be able to selective turn on or off blocks of connections, for example turning off the **X_1_**-to-**voc** connections and on the **X_2_**-to-**voc** connections when the goal is to vocalize the color of the object currently being viewed by spotlight #2.

Such a system appears to solve the immediate task, but runs the risk of violating “role-filler independence” (Hummel et al., 2004; Hummel, 2011) if ever the training of direct connections to **voc** creates a situation where **voc** can understand a color represented by **X_1_** but not **X_2_** or vice versa. This realization brings us to the first part of the DPAAN solution – in order to avoid violations of role-filler independence we should make sure that the training is synchronized such that whenever **voc** learns the translation for a concept used by **X_1_**, **voc** should simultaneously learn the translation for the same concept used by **X_2_**. Given the Multiple Slots Multiple Spotlights visual model depicted in Figure 1 the solution to this is relatively simple – only perform such training of direct connections to **voc** when spotlight #1 and spotlight #2 are locked on the same object. This presents a preliminary answer to question #2 above – there is no need to “transfer” the associations learned on the **X_1_**-to-**voc** connections to the **X_2_**-to-**voc** connections if these associations are always kept synchronized by simultaneous training while looking at the same object.

This “solution” however is not very scalable in that it puts a tremendous burden on the training of the many connections to **voc** which must not only be able to vocalize the colors represented in **X_1_** and **X_2_** but must also vocalize the shapes represented in **Y_1_** and **Y_2_** which (in the “world languages” analogy) should themselves be considered as speaking yet different languages (e.g., Chinese and Japanese). Further, in any realistic model of the brain, like the one proposed in the ACT-R theory described below, there exists many dozens of different perceptual, motor, and cognitive modules many of which must communicate symbols with each other despite the fact that each speaks a different neural language. This solution would suggest that each of these modules must become a “language savant” fluent in the dozens of distinct dialects of the other modules it must communicate with. Further, the training of the connections between all of the modules would have to be kept synchronized for each new vocabulary word or else there would be violations of role-filler independence.

The DPAAN solution presented in this paper overcomes these difficulties by positing a central DPAAN which serves as a “universal translator” between all the various modules of the brain. The DPAAN network is a fully connected autoassociative memory having a set of stable attractor states. Each of these stable attractor states acts as a symbol in a “universal language,” and all the other modules’ languages are translated into this universal language facilitating between-module communications. Each module is connected to a specific part of the global DPAAN via the module’s own trainable association network which serves the task of translating the module’s specific language to the universal language of the global DPAAN or vice versa.

When a new symbol is to needed to be learned (e.g., when a mother holds a red object in front of a baby for the first time and says “red”) an unused stable state in the DPAAN is activated and all the modules train their association networks based on that global attractor state. This training associates each individual module’s neural pattern for “red” with a specific *piece* of the global DPAAN’s stable attractor state which can be thought of as the universal language’s symbol for “red.” This is the key conceptual step of the DPAAN formulation where a symbol is no longer thought of as represented by a unique pattern of activation but is instead thought of as a *piece* of a global stable attractor state. We will see that this is the key to achieving roll-filler independence in an anatomical binding framework.

In operation (as opposed to during training) the DPAAN must serve as a “global workspace” and “global switchboard” between modules allowing them to communicate dynamically and without crosstalk. The DPAAN achieves this by being “dynamically partitionable”. During training all of the DPAAN’s synapses are turned on (i.e., set to their autoassociative memory weights) ensuring that there is one global stable state for the synchronized training of each module’s association network. But during operation, blocks of the DPAAN’s synapses are turned off thus effectively dividing the DPAAN into many independent autoassociative memory buffers – one for each of the modules. A switchboard-like connection between two modules (say A and B) is created when the synapses projecting from buffer A of the DPAAN to buffer B are turned back on. This turning on of a particular block of the DPAAN’s synapses reconstitutes part of the global autoassociative memory network driving buffer B into part of the same global attractor state that buffer A is currently in. We will, see below that this serves the task of simultaneously transferring and translating the symbol from module A to module B. Finally, the semantic contents of different modules’ buffers can be compared for equality by calculating something like the Hopfield energy function (Hopfield, 1982) over the synapses connecting the separate partitions of the DPAAN – in effect asking if the patterns of activation in the two buffers are actually different pieces of the same global stable state.

In the section “Example #1: Use of a DPAAN to Solve the Perceptual Binding Problem” below we will see how these two properties (the ability to transfer semantic contents between the modules’ buffers and the ability compare the semantic contents of two buffers for equality) are all that is needed to address the two questions above in relation to the visual binding model. In later sections we will, see how such a central DPAAN, acting as a universal translator^5^ and switchboard between brain modules, represents a biologically plausible hypothesis for how the brain performs complex syntactic operations.

## Results

### Formal definition of a dynamically partitionable autoassociative network

A DPAAN consists of *K* sets of neurons called partitions (or slots) where in general each partition can contain a different number of neurons *N*_1_, *N*_2_, …, *N_K_*. We will designate the activation of any particular partition as the vector **x***_k_*, where *k* ∈ 1, 2, …, *K*. The entire DPANN network therefore contains $N=\Sigma_{k=1}^{K}N_{k}$ neurons whose total activation vector will be denoted by the concatenated vector **x** = [**x**_1_; **x**_2_; …; **x***_K_*] and whose individual neural unit activations will be denoted by *x_i_* where *i* ∈ 1, 2, …, *N*. The DPAAN is a fully connected autoassociative network with weights *w_ij_* where *w_ij_* is the weight to postsynaptic unit *i* from presynaptic unit *j*. These weights are trained by learning rules like Hopfield, Hebbian, etc., to contain several stable attractor states.

Let the network be globally trained with a total of *L* patterns (i.e., *L* stable attractors) designated ***P****^l^* where *l* ∈ 1, 2, …, *L*. That is, when the network falls into stable state ***P****^l^* then $x_{i}=p_{i}^{l}$ for all *i* ∈ 1, 2, …, *N*. These *L* patterns form the basic symbol vocabulary of the DPANN and each pattern is meant to have a unique semantic meaning, for example ***P***^1^ might mean “red” to the system, and ***P***^2^ might mean “blue.” More crucially, if a particular slot **x***_k_*, has an activation that coincides with the activation that these same neurons would have if the total system were in the stable attractor ***P****^l^* (call this activation on the *k*th slot ${\text{P}}_{k}^{l}$) then we say that that particular slot is representing the symbol *l* irrespective of what activation the rest of the network has. That is, if pattern ***P***^2^ means “blue,” and the third slot has activation ${\text{x}}_{\text{3}}={\text{P}}_{\text{3}}^{\text{2}},$ then we will claim that slot 3 is representing the symbol “blue.” Also, if the fifth slot has activation ${\text{x}}_{5}={\text{P}}_{\text{5}}^{\text{2}}$ then we will claim that slot 5 is also representing the symbol “blue” even though the vectors ${\text{P}}_{\text{3}}^{\text{2}}\;\text{and}\;{\text{P}}_{\text{5}}^{\text{2}}$ are not mathematically equal nor even guaranteed to have the same number of elements. This claim of semantic compatibility will be justified below.

The DPAAN is “dynamically partitionable” by setting to zero different subsets of weights while keeping all other synapses at the same values they were trained at. For example, if we set to zero all synapses that connect two neurons that are in different partitions but leave constant all synapses that connect two neurons in the same partition then we effectively split the original autoassociative network into *K* subnetworks. The key thing to note is that each of these subnetworks is still autoassociative and (given sufficient size) each still contains the *L* stable states trained within the original network. What this means is that a DPAAN partitioned into *K* separate slots can act somewhat like a set of memory buffers in a computer, each slot can be put into any of its stable states and remain there.

To truly make the slots in a DPAAN behave like the buffers in a computer we would like to be able to perform two crucial operations on them. These operations are:

1. Slot-to-slot transfer
2. Slot-to-slot equality detection

As discussed previously, these operations are trivial in a computer because it is built to maintain a one-to-one correspondence between the bits in each and every buffer. For the DPAAN the definition of transfer and equality will be slightly different. We will say that the symbol in slot 1 has been successfully transferred to slot 2 if before the transfer slot 1 contained the activation pattern ${\text{x}}_{\text{1}}={\text{P}}_{\text{1}}^{l},$ and after the transfer slot 2 now contains the pattern ${\text{x}}_{\text{2}}={\text{P}}_{\text{2}}^{l}.$ More generally, we will say that the DPAAN as a whole provides semantic consistency for all slot-to-slot transfers if and only if for all *k*, *k*′, and *l*, if ${\text{x}}_{k}={\text{P}}_{k}^{l}$ is the activation of slot *k*, then a transfer operation from slot *k* to slot *k*′ will yield: ${\text{x}}_{k^{\prime }}={\text{P}}_{k^{\prime }}^{l}.$ In words, a transfer from slot *k* to slot *k*′ is successful if after the transfer the *k*′ partition is now in a part of the same global attractor state that the *k* partition was in.

The DPAAN is built to ensure such semantic consistency for all slot-to-slot transfers because it is trained as a single autoassociative network. To transfer the contents from slot 1 to slot 2 one simply blanks the contents of slot 2 and then turns on any synapses projecting from slot 1 neurons to slot 2 neurons (but leaves zero all synapses projecting from slot 2 to slot 1). This turning on of synapses between the two slots reconstitutes part of the original autoassociative network. Turning on only the synapses projecting from slot 1 to slot 2 is equivalent to clamping the contents of slot 1 in its current state while letting slot 2’s neurons evolve toward a combined local minimum. The result is that the combined slots evolve toward the closest originally stored pattern which will be a “completion” of the pattern stored in slot 1. All slot-to-slot transfers take this form. To transfer the contents of slot *k* to slot *k*′ one simply blanks the current contents of slot *k*′ and then turns on the synapses projecting from the neurons of slot *k* to the neurons of slot *k*′. After the system evolves to a combined stable state, the between slot synapses are once again turned off but the within-slot synapses remain on, thus storing the transferred pattern.

Slot-to-slot equality detection requires that there be a set of neurons somewhere outside the core DPAAN which signal if the contents of slot *k* is semantically equal to the contents of slot *k*′. That is, there should be a set of neurons eq*_kk_′* (one for each unique pair of slots) which have the following activation function:

$$\text{Let }{\text{x}}_{\text{k}}={\text{P}}_{k}^{l}\;\text{ and }\;{\text{x}}_{k^{\prime }}={\text{P}}_{k^{\prime }}^{l^{\prime }}\;\text{ then }\;\text{e}{\text{q}}_{kk^{\prime }}=\left\{\;\begin{array}{ccccc} 1 & \text{if} & l & = & l^{\prime }\\ 0 & \text{if} & l & \neq & l^{\prime } \end{array}\right\}$$

There are several ways such neurons could be trained during the initial training of the network, but the simplest is to create neurons that are in some way sensitive to the autoassociative network’s energy function calculated over the set of weights between slot *k* and slot *k*′. For example, if the DPAAN network was trained with the Hopfield rule then the Hopfield energy function for the entire network would be:

$$E=-\frac{1}{2}\underset{i}{\sum }\left(\underset{j\neq i}{\sum }w_{ij}x_{i}x_{j}\right)$$

This energy function^6^ is guaranteed to be low for the trained patterns ***P****^l^* where *l* ∈ 1, 2, …, *L*. Now we can define a new energy function that is computed only over the set of synapses projecting from slot *k* to slot *k*′ and vice versa (the expression could of course be simplified since a Hopfield network has symmetric synapses):

$$E_{kk^{\prime }}=-\underset{i\;\in \;\text{slot}\;k}{\sum }\left(\underset{j\;\in \;\text{slot}\;k^{\prime }}{\sum }w_{ij}x_{i}x_{j}\right)-\underset{j\;\in \;\text{slot}\;k}{\sum }\left(\underset{i\;\in \;\text{slot}\;k^{\prime }}{\sum }w_{ij}x_{i}x_{j}\right)$$

This expression is guaranteed to be low precisely in those cases where slot *k* and slot *k*′ have activations that are part of the same original trained pattern. Thus thresholding this function can create the set of equality detection neurons we are looking for.

$${\text{eq}}_{kk^{\prime }}=\left\{\begin{array}{c} 1\text{ if }E_{kk^{\prime }}<\text{Treshold}\\ 0\text{ otherwise} \end{array}\right\}$$

It remains to be explored how such neurons could be trained in a biologically plausible manner.

### Example #1: Use of a DPAAN to solve the perceptual binding problem

I will now describe a simple model that demonstrates the DPAAN’s ability to overcome the previous objections put forward against the anatomical binding hypothesis. Recall the two central arguments that were put forward with reference to Figure 1:

1. How could the larger neural system determine whether object #1 and object #2 had the same or different colors (or shapes, etc.)?
2. How could the system vocalize the color of the object represented by one set of neurons by making use of the associations learned on a different set of neurons?

**Figure 2:**
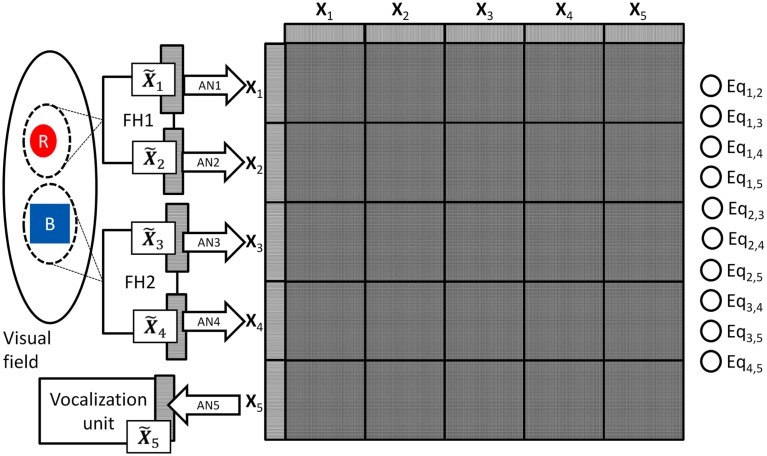
**Modification of Figure 1’s vision model to incorporate a DPAAN network**. FH1’s color and shape output fields have been relabeled as ${\tilde{\text{X}}}_{1}\;\text{and}\;{\tilde{\text{X}}}_{2}.\;$ Specific patterns of activity on these neural fields are associated with the corresponding DPAAN slots **X_1_** and **X_2_** via trainable association networks AN1 and AN2. Similarly, FH2’s color and shape fields ${\tilde{\text{X}}}_{3}\;\text{and}\;{\tilde{\text{X}}}_{4}\;$ are associated with the corresponding DPAAN slots **X_3_** and **X_4_** via trainable association networks AN3 and AN4. Neural field ${\tilde{\text{X}}}_{5}$ drives a vocalization unit to produce audible responses. This unit is itself driven by the contents of DPAAN slot **X_5_** via trainable association network AN5.

To see how the DPAAN addresses both of these questions we will redraw Figure 1 to include a DPAAN with five partitions corresponding to the five original sets of neurons **X_1_**, **X_2_**, **Y_1_**, **Y_2_**, and **voc** (see Figure 2), but to maintain consistency with the DPAAN’s formal definition above we will re-label the sets of neurons as **X_1_** … **X_5_**; that is, **X_1_** and **X_2_** will serve to represent the color and shape respectively of the object highlighted by attentional spotlight #1. Neural sets **X_3_** and **X_4_** will serve to represent the color and shape of the object highlighted by attentional spotlight #2, and **X_5_** will be the **voc** neurons which must learn how to vocalize various object attributes.

**Figure 3:**
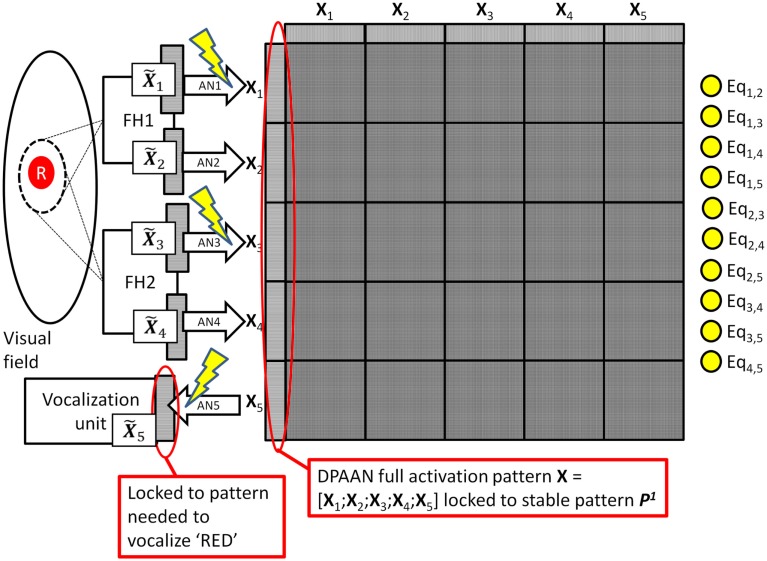
**System learning the symbol for red**. Lightning bolts designate which association networks (AN1, AN3, and AN5) are being trained during this procedure. Note that the equality detection neurons are all on (yellow) since the pattern stored across all partitions is a single stable state.

Assume that the DPAAN’s weights are already pre-trained with a number of stable patterns ***P***^1^, ***P***^2^, etc. One can think of these as ready-made symbols which have not acquired any semantic meaning yet because they have not acquired any associations with the outside world (i.e., they have not been “grounded”). The system using the DPAAN will learn to associate different object properties with these patterns in the following way: During the training of the system for colors, a red circle is presented and the system locks *both spotlights* onto this single object. As we will, see, it is crucially important that during all training the system locks *both spotlights* onto a single object. FH1 and FH2 process the object and produce neural outputs $\left({\tilde{\text{X}}}_{1},\;{\tilde{\text{X}}}_{2},\;{\tilde{\text{X}}}_{\text{3}},\;{\tilde{\text{X}}}_{4}\right)$ representing the color and shape of the object. Because this is a new color, the system chooses an unused stable pattern in the DPAAN (say ***P***^1^) and locks the network into this state. This pattern ***P***^1^ will eventually come to mean “red” to the system. Next the instructor locks a pattern of activation into the vocalization unit’s neurons $\left({\tilde{\text{X}}}_{\text{5}}\right)$ that will result in the correct vocalization of the word “red.” Now the association network AN1 (connecting ${\tilde{\text{X}}}_{1}\;to\;{\text{X}}_{1}$) and the association network AN3 (connecting ${\tilde{\text{X}}}_{3}\;to\;{\text{X}}_{3}$) and the association network AN5 (connecting ${\text{X}}_{5}\;to\;{\tilde{\text{X}}}_{5}$) are trained. This training of association networks AN1 and AN3 will allow any future observation of a red object (either by FH1 or by FH2) to be correctly associated with the pattern ***P***^1^. Also, this training of association network AN5 will allow the vocalization unit to correctly pronounce the word “red” whenever the pattern ***P***^1^ is held in the DPAAN. Because the system is learning a color, the association networks for shape (i.e., AN2 and AN4) are left alone. This training procedure for the color “red” is depicted in Figure 3.

The same training procedure is repeated with different colors. For example, a blue object is presented and both spotlights lock onto it producing neural representations for blue on ${\tilde{\text{X}}}_{1}\;\text{and }{\tilde{\text{X}}}_{3},$ and the instructor puts a pattern on the vocalization unit’s neurons (${\tilde{\text{X}}}_{5}$) that will result in the correct vocalization of the word “blue.” A new unused pattern (say ***P***^2^) is locked into the DPAAN and the appropriate association networks (AN1, AN3, and AN5) are again trained. The system now has learned the symbol for “blue.” The process is repeated for all other basic level symbols (e.g., “square” by training association networks AN2, AN4, and AN5 with the pattern ***P***^3^ locked into the DPAAN, and “circle” by training the association networks AN2, AN4, and AN5 with the pattern ***P***^4^ locked into the DPAAN). At the end of these four training steps the network will have learned the following DPAAN symbol associations: ***P***^1^ = “RED,” ***P***^2^ = “BLUE,” ***P***^3^ = “SQUARE,” ***P***^4^ = “CIRCLE.” From here on we will refer to these four DPAAN stable patterns as ***P***^RED^, ***P***^BLUE^, ***P***^SQUARE^, and ***P***^CIRCLE^.

**Figure 4:**
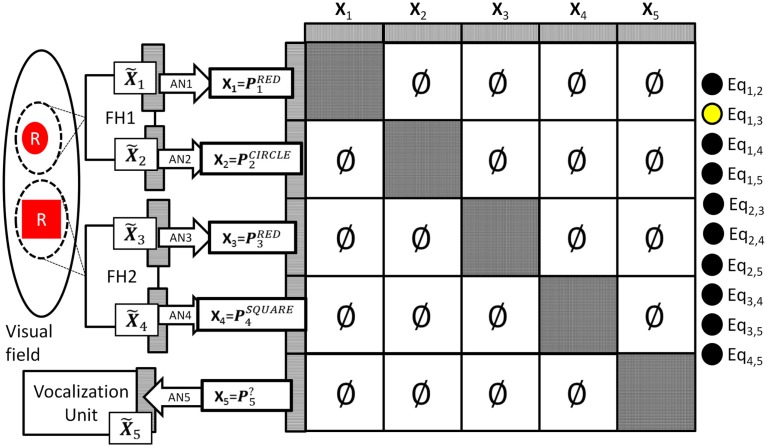
**System representing a visual scene containing a red circle and a red square**. Notice that all between slot DPAAN weights have been set to zero so that they can store symbols independently. Also notice that equality detection neuron Eq_1,3_ is active signaling that the contents of DPAAN slots **X_1_** and **X_3_** are semantically equal (both signaling the symbol “red”).

During operation the DPAAN’s synaptic weights are normally set such that each partition (slot) acts as an independent autoassociative network, and spotlight #1 and #2 are free to lock onto different objects. Let’s say that the system locks spotlight #1 onto a red circle and it locks spotlight #2 onto a red square. This situation is depicted in Figure 4.

In this situation the association learned by AN1 will ensure that slot **X_1_**’s activation is set to $P_{\text{1}}^{\text{RED}}.$ That is, slot **X_1_**’s neurons will fall into the activity pattern that is the subset of the ***P***^RED^ pattern that covers the **X_1_** neurons. The other slots driven by FH1 and FH2 will similarly fall into their appropriate associated patterns, notice however that because the DPAAN weights between slots have been set to zero there is no interference from slot-to-slot, they are each free to fall into whatever is the closest associated pattern. In this example, slots **X_1_** and **X_3_** happen to be set to different subsets of the same original global stable state, namely ***P***^RED^. This means that the energy function calculated over the synapses between these sets of neurons will be low, and thus the equality detection neuron whose output is based on this energy calculation (Eq_1,3_) will fire (depicted in the figure as a yellow filled circle). No other equality detection neurons will fire in this example (depicted in the figure as black filled circles).

This example provides an answer to our first question: “How could the larger neural system using this anatomical binding scheme determine whether object #1 and object #2 had the same or different colors (or shapes, etc.)?” One can, see that the equality detection neurons are doing just that, and will generalize correctly over all learned patterns. In the above example Eq_1,3_ will fire signaling to the system that the two objects have the *same* color, and Eq_2,4_ will not fire signaling that the two objects have *different* shapes.

**Figure 5:**
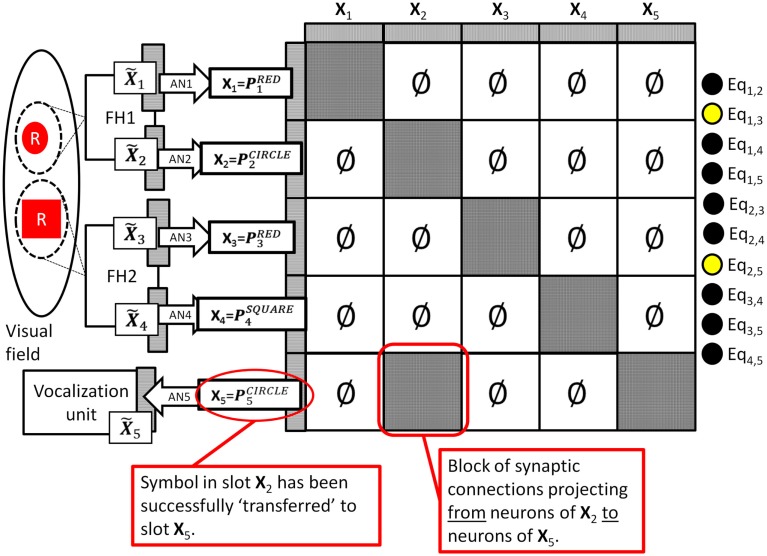
**Diagram showing how the DPAAN’s dynamic partitions are used to “transfer” the semantic contents of slot X_2_ to slot X_5_ (by momentarily turning on the synapses connecting slot X_2_ to slot X_5_)**. This allows the system to vocalize the shape of the object highlighted by spotlight #1. Similar transfers allow for any other perceptual attribute of either object to be vocalized.

What about our second question: “How could the system vocalize the color of the object represented by one set of neurons by making use of the associations learned on a different set of neurons?” In the above example the ability to vocalize was trained into association network AN5 connected to slot **X_5_**. To vocalize a particular perceptual attribute the system must transfer the symbol from one of the perceptual slots (**X_1_**, **X_2_**, **X_3_**, **X_4_**) to this motor vocalization slot (**X_5_**). Recall that such transfers are trivial to perform between computer buffers which are built to have a one-to-one correspondence between bits, but they have often been assumed impossible to perform in a biologically wired brain. The DPAAN handles such transfers in a novel way; instead of transferring the exact firing pattern from one set of neurons to another it instead reestablishes (by un-zeroing blocks of synapses) the part of the original autoassociative network that connects these two slots. Let’s say we wanted the network to vocalize the shape of the object in spotlight #1. The symbol for this shape is stored in slot **X_2_** as ${\text{P}}_{2}^{\text{CIRCLE}}.$ To perform this transfer the contents of the target slot (**X_5_**) are “blanked” and then the block of synapses that connects the neurons in slot **X_2_** with the neurons in slot **X_5_** is momentarily un-zeroed. This is depicted in Figure 5 below.

The consequence is that the neurons in slot **X_5_** are driven toward the stable state dictated by the pattern in slot **X_2_**. The result being that slot **X_5_** will be driven toward completion of the pattern stored in ${\text{X}}_{2}\left(P_{5}^{\text{CIRCLE}}\right)$ and thus will end up in a state of activation corresponding to pattern ${\text{X}}_{\text{5}}\text{ = }{\text{P}}_{5}^{\text{CIRCLE}}.$ This is precisely the pattern that association network AN5 was trained on to generate the vocalization of the word “circle.” Such directed transfers from any one of the perceptual slots (**X_1_**, **X_2_**, **X_3_**, **X_4_**) to the motor vocalization slot will allow the system to vocalize any particular perceptual attribute.

#### Simulation results

A simulation was written to demonstrate the DPAAN’s key new features of slot-to-slot equality detection and slot-to-slot transfer. Complete Matlab simulation files are provided in the supplementary online material.

The program first defines a DPAAN comprising five slots containing 100 neurons each. The DPAAN’s slots are meant to correspond to the five slots depicted in Figure 2. Next the program defines the symbol vocabulary of the DPAAN by first creating random neural activation vectors corresponding to the symbols “red,” “blue,” “square,” “circle.” These random neural activation vectors are used to generate the DPANN weight matrix via the Hopfield learning rule (Anastasio, 2010), thus creating a stable attractor for each symbol.

Initial activation of the DPAAN is set to reflect Figure 4, representing the state the network would achieve after perceptually processing a scene containing a red square and a red circle. (The operation of the feature hierarchies FH1 and FH2 and association networks AN1–4 needed to achieve this initial state were not incorporated into this simulation since other simulations have been published showing how this can be performed, e.g., Serre et al., 2005; Hayworth, 2009). Initial activation of slot **X_5_** neurons is set to zero. The weight matrix of the DPAAN is masked leaving all synapses which connect two neurons in the same slot to their Hopfield trained value, and setting all synapses connecting neurons in separate slots to zero except for those synapses which project from slot **X**_1_ neurons to slot **X_5_** neurons. This masking of the DPAAN’s weight matrix directs the DPAAN to retain the current contents of slots **X_1_** through **X_4_**, and to transfer the contents of slot **X_1_** to slot **X_5_**.

**Figure 6:**
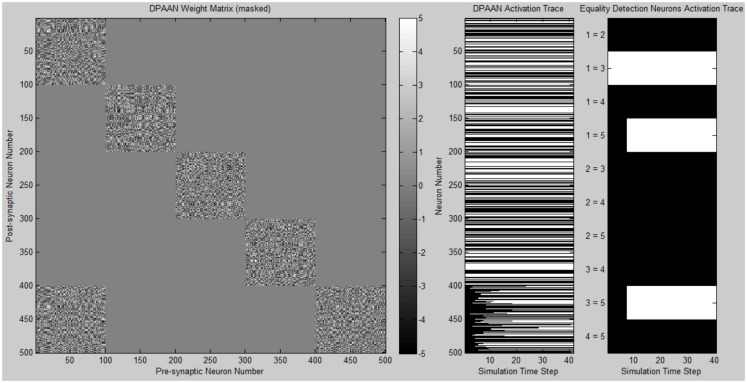
**Plot of simulation results showing the masked DPAAN weight matrix, a time trace of the DPAAN neural activation vector, and a time trace of the equality detection neurons’ activations throughout the simulation**. (White = active neuron, black = inactive neuron).

Figure 6 shows a plot of the masked DPAAN weight matrix, a time trace of the DPAAN neural activation vector, and a time trace of the equality detection neurons’ activations throughout the simulation. At start of simulation the part of the DPANN activation vector corresponding to slot **X_5_** (neurons 401–500) shows zero activation, but this changes over the course of the simulation due to the influence of synapses projecting from **X_1_**. The semantic contents of **X_1_** are being transferred to **X_5_** via the influence of these non-masked synapses projecting from **X_1_** to **X_5_**. The activation patterns of slots **X_1_**, **X_2_**, **X_3_**, and **X_4_** remain constant throughout the simulation due to the fact that the initial state of the network corresponded to stable states of these four subnetworks. Only Eq_1,3_ (the equality detection neuron which compares the contents of **X_1_** to **X_3_**) is active at the beginning of the simulation. The Eq_1,3_ neuron is signaling that the semantic contents of slot **X_1_** and slot **X_3_** are the same – namely they both contain the symbol “red.” Recall that the activation state of an equality detection neuron is based on the Hopfield energy function calculated over the synapses connecting the two slots. During the course of the simulation Eq_1,5_ and Eq_3,5_ also become active reflecting that the symbol “red” has been successfully transferred to **X_5_**. At the end of the simulation the equality detection network correctly reflects that slots **X_1_**, **X_3_**, and **X_5_** all contain the same symbol.

### Example #2: Modeling syntax-sensitive rule-based decisions using a DPAAN

As described in the introduction, cognitive modeling has traditionally assumed not only that syntax can be represented but that it can be recognized and manipulated as well. A classic example is the following:

John loves Mary. Mary loves Sam. John is jealous of who?

A system that understands the rules of jealousy would be able to correctly replace the “who” with “Sam.” Importantly, this system must generalize this rule only where appropriate allowing it to apply to all people. Below is a characterization of the jealously rule in a format using variables:

If (**X_1_** loves **X_2_**) and (**X_3_** loves **X_4_**) and (**X_5_** is jealous of **X_6_**) and
(**X_1_** = **X_5_**) and (**X_2_** = **X_3_**) and (**X_1_** ≠ **X_4_**)& (**X_6_** = “*who*”)
Then (**X_4_** → **X_6_**)

In the above example the variables would be initialized as follows: **X_1_** = “John,” **X_2_** = “Mary,” **X_3_** = “Mary,” **X_4_** = “Sam,” **X_5_** = “John,” **X_6_** = “who.” This satisfies the if-clause leading to execution of the then-clause, namely the semantic contents of variable **X_4_** will be transferred to **X_6_**. This transfer answers the question, signaling that John is in fact jealous of Sam.

This type of variablized and equality-conditioned rule is the bread-and-butter of classical AI systems but it has been notoriously difficult to train neural systems to implement such rules precisely because of the neural binding problem. In contrast, the DPAAN framework has been designed specifically to make such rule-based operations possible.

**Figure 7:**
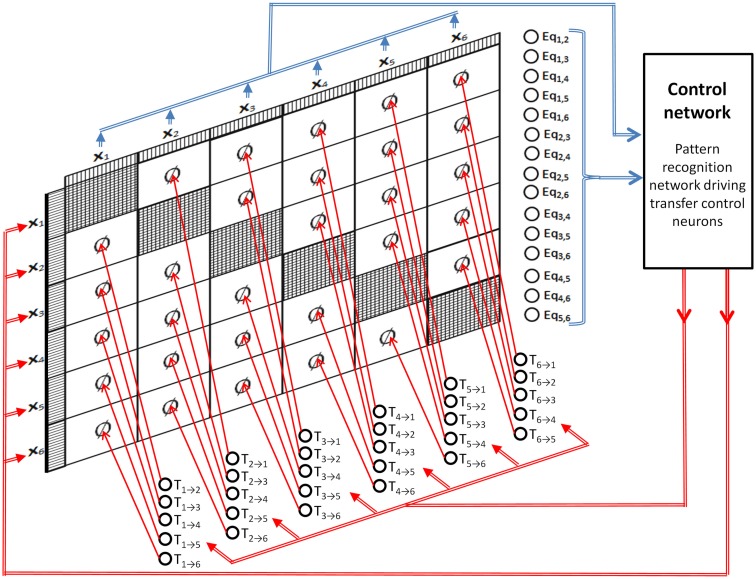
**Dynamically Partitionable AutoAssociative Network and control network for implementing syntax-sensitive rule application**.

Figure 7 shows a DPAAN with a set of transfer control neurons (T_1 → 2_, T_1 → 3_, etc.) which are used to turn on or off blocks of synapses in the DPAAN (these neurons thus control the dynamic partitioning of the network). Figure 7 shows a control network receiving inputs from all of the DPAAN slots and from its equality detection neurons. This control network sends outputs to the transfer control neurons and can itself overwrite any of the DPAAN slots. This control network looks complicated but it is actually only a pattern recognition network – i.e., it maps one neural pattern to another. If the control network recognizes a particular pattern on the DPAAN slots and the equality detection neurons then it will output its matched stored pattern to the transfer control neurons and will possibly overwrite some of the DPAAN slots. This control network is thus able to orchestrate transfers between slots in the DPAAN, and this orchestration is contingent on the equality (or non-equality) between DPAAN slots. This is precisely what is needed to implement rule-based decisions like the Jealousy rule above.

**Figure 8:**
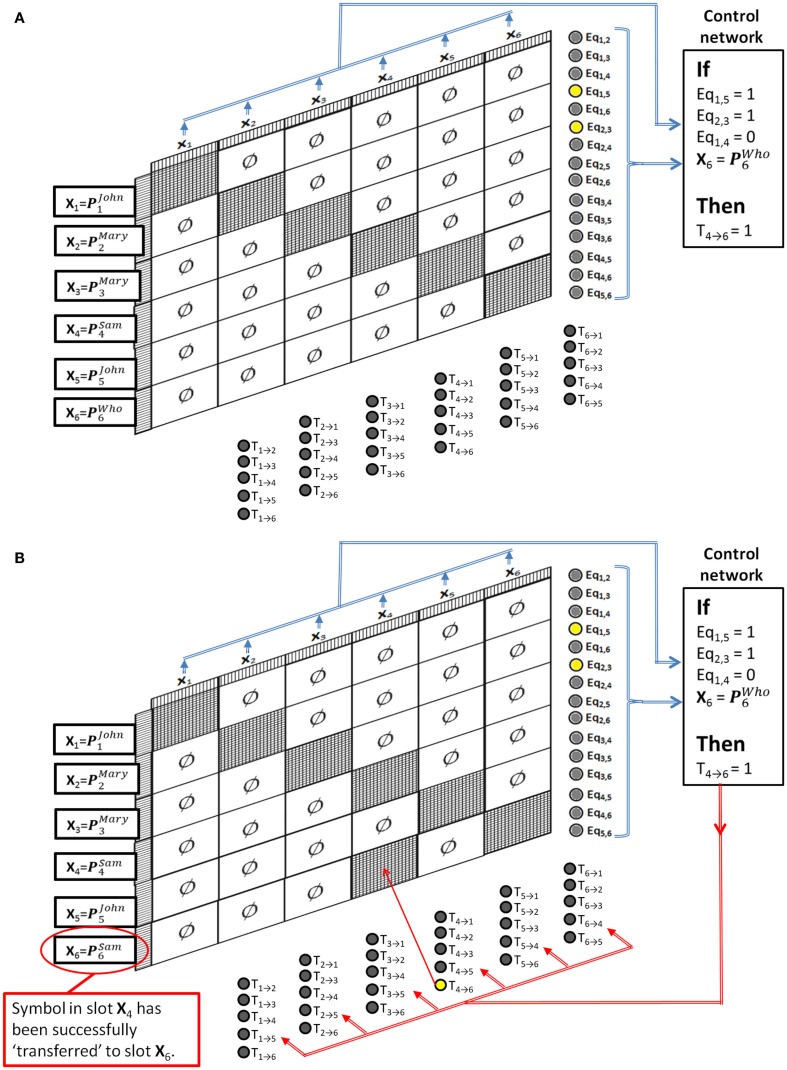
**Dynamically Partitionable autoassociative network and control network solving the Jealously problem**. **(A)** Pattern recognition part of the cycle, **(B)** rule application part of the cycle.

To see how this can solve the Jealousy problem, first assume that the system has already been trained to have several stable states ***P***^1^, ***P***^2^, etc., and that these have been associated with individual names of people (perhaps through a perceptual association network like the ones in the previous example). Then we can refer to these stable states as ***P***^John^, ***P***^Mary^, ***P***^Sam^, ***P***^Who^, etc. The control network for implementing the single Jealously rule will simply check that neurons Eq_1,5_ and Eq_2,3_ are firing, and that neuron Eq_1,4_ is *not* firing, and that the pattern in slot ${\text{X}}_{6}={\text{P}}_{6}^{\text{Who}}.$ If this pattern is seen then the control network should output a pattern that simply turns on the transfer control neuron T_4 → 6_. Figure 8A shows the pattern recognition part of this cycle, and Figure 8B shows the activity after the pattern is recognized. The result is that the semantic contents of slot 4 (“Sam”) is transferred to slot 6, thus successfully applying the Jealousy rule.

This network will correctly apply the rule no matter what symbol names are in the slots, and it will not apply the rule in situations in which the syntax is inappropriate. For example, if John loves Mary and Mary loves John then John is not jealous at all. The network understands this because it tests for Eq_1,4_ = 0. Also, any number of rules could be stored in the control network simultaneously. Whichever rule matches the DPAAN’s current state will be the rule that is applied.

### Simulation results

A simulation was written to demonstrate how a DPAAN attached to a control network could be used to implement the Jealously rule. Matlab simulation files are provided in the supplementary online material.

The simulation program first defines a DPAAN comprising six slots containing 100 neurons each. These are meant to correspond to the six slots depicted in Figure 8 above. A symbol vocabulary is created for the DPAAN by creating a set of random neural activation vectors, one for each of the symbols “John,” “Mary,” “Sam,” “Leela,” “Fry,” “Zap,” “Kif,” “Amy,” and “who.” These are used to generate the weight matrix (via the Hopfield rule) for the DPANN, thus creating a stable attractor state for each symbol.

**Figure 9:**
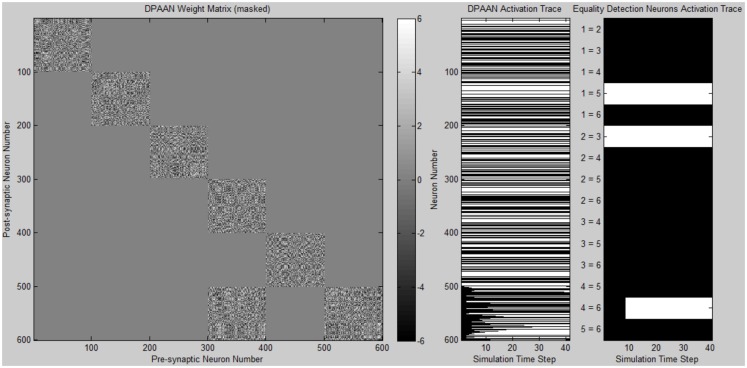
**Simulation results for the Jealously problem example**. (White = active neuron, black = inactive neuron).

The if-clause of any rule simply requires testing for a particular pattern of activity across the equality detection neurons and across the DPAAN slots. The Jealously rule in particular requires testing if the pattern for “who” is present in **X_6_**. In the simulation a network of “symbol detection neurons” was created based on simple perceptron matching. One of these symbol detection neurons signals if the activation pattern representing the symbol “who” is present in slot **X_6_**. We will call this particular symbol detection neuron SD_6, “who”_. The test for the Jealously rule activation then amounts to testing if neurons Eq_1,5_ and Eq_2,3_ are firing, that neuron Eq_1,4_ is *not* firing, and that SD_6, “who”_ is firing. If this condition is met then the transfer control neuron T_4 → 6_ is activated turning on the synapses projecting from **X_4_** to **X_6_**. This logic is implemented in the simulation to control the DPAAN, ensuring that the block of synapses projecting from **X_4_** to **X_6_** is only activated if all the conditions of the Jealously rule are satisfied. Figure 9 shows the results of one simulation in which all of the conditions are met. The program in the supplementary material includes several other test cases verifying that the rule is activated only when appropriate.

### Review of rule-based operation using a DPAAN

Let’s take a moment to review how the DPAAN was used to implement this rule-based functionality in order to make clear that any set of similar rules can be implemented in a single DPAAN in this same manner. First a DPAAN is created having *K* slots. The DPAAN’s synaptic matrix is then trained to contain *L* stable attractor states, each state corresponding to one of the *L* symbols comprising the DPAAN’s vocabulary. A set of equality detection neurons is then created, one neuron for each unique pair of slots [for a total of *K*(*K* − 1)/2 neurons]. Then a set of symbol detection neurons (simple perceptrons) is created, one neuron for each slot + symbol combination that the rules need to explicitly test for. Finally a set of transfer control neurons is created, one for each block of synapses projecting between slots [a total of *K*(*K* − 1) transfer control neurons].

**Figure 10:**
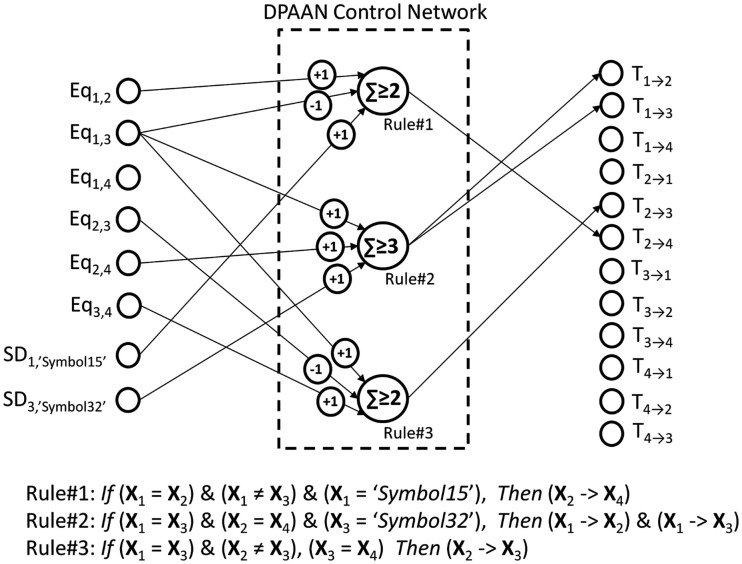
**A DPAAN control network (consisting of three neurons) which is designed to implement the three rules shown**.

Given this architecture, any set of if-then rules of the type described above can be implemented. Each rule is implemented as a single linear summing, thresholded neuron receiving synapses from specific equality detection neurons and from specific symbol detection neurons, and sending its activation to specific transfer control neurons. Figure 10 shows how this works, depicting a DPAAN control network consisting of three neurons designed to implement the three rules written algebraically in the figure.

In operation, the DPAAN’s slots are initialized with activation patterns corresponding to any of the learned symbols of the system. In a feed forward manner, this DPAAN activation drives the equality detection neurons and symbol detection neurons, and these in turn drive the rule neurons. If any of the rule neurons exceeds its threshold then it will in turn activate one (or more) of the transfer control neurons which will turn on a whole block of synaptic connections in the DPAAN. At this point, simulation of the DPAAN’s dynamics will cause the semantic contents of one slot to be transferred to another. This full operation cycle illustrates how such DPAAN-based rule functionality can be sensitive to syntax (through the equality detection neurons which are *blind* to the actual contents of the slots being compared) and can elicit syntax-based changes (by directing transfer between slots again *blind* to the actual content of the slot whose contents is transferred).

### Example #3: Using a DPAAN as the core of a neural implementation of ACT-R

I assert that the DPAAN operation described above is a completely general solution to the classic neural binding problem. One way to demonstrate this is to show how the core part of the cognitive architecture ACT-R could be implemented by a DPAAN.

ACT-R is the most advanced and experimentally well supported cognitive model of the human mind available today. The ACT-R architecture has been used to model everything from basic stimulus response timing, and visual search, to problem solving, language understanding, and skill acquisition (http://act-r.psy.cmu.edu/). It would be impossible to cover the entire ACT-R architecture in detail here, but a basic overview is necessary in order to understand how I am proposing that a DPAAN can be used as an implementation of ACT-R’s global workspace.

#### Brief overview of ACT-R

**Figure 11:**
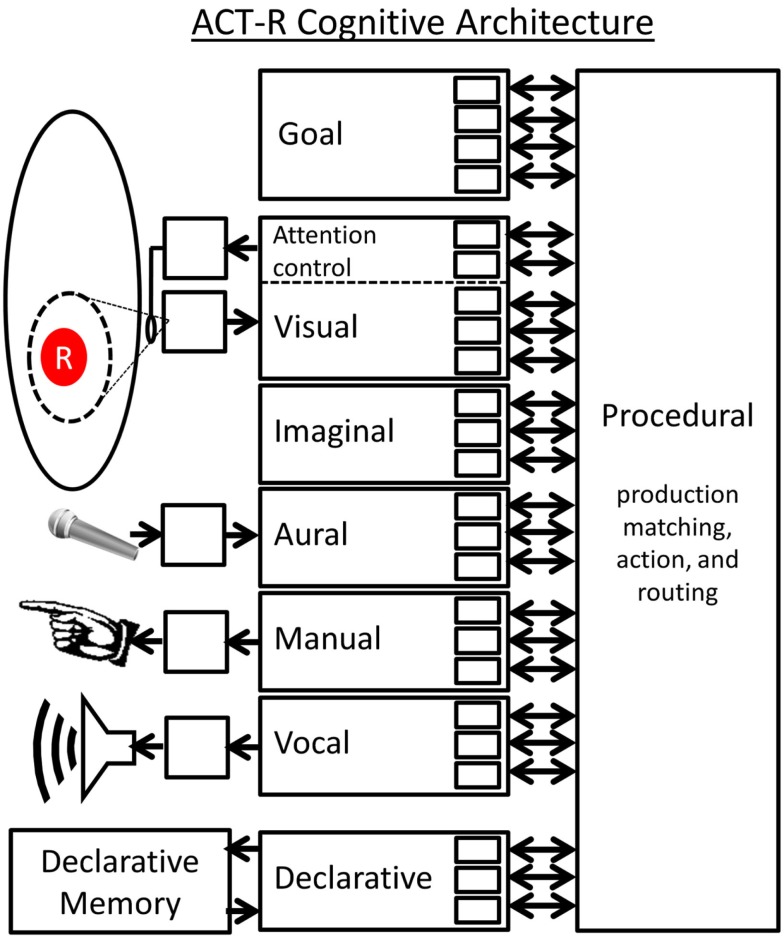
**Schematic representation of the ACT-R architecture**.

ACT-R (Figure 11) posits that the brain is composed of several modules. Each module is unique in how it performs its function but all modules have a common interface to the brain’s central Procedural module. This common interface is that each module has a set of slots each of which can contain one symbol. A module’s interface slots can be written to by the module itself and/or by the Procedural module. The collection of all of these slots is the “global workspace” of the ACT-R system. There is one special module called the Declarative Memory (DM) module which is connected to a memory store. The Procedural module will fill in some of the slots of the DM module but leave other slots blank, and will request that the blank slots be filled in by associative recall. As such, each individual DM “chunk” is a list of particular symbols.

The heart of ACT-R is the Procedural module which stores a set of “production rules” which are simply if-then rules like the one described in the Jealously problem above. The if-clause of a production rule may require that a particular slot is filled with a certain symbol. It may also require that two slots contain the same (or different) symbols. As such, the if-clause is simply a list of slot-filler constraints and slot equality constraints. If these if-clause conditions are met, then the production will “fire” performing the actions specified by its then-clause. Then-clause actions come in three forms: (1) Writing a particular symbol to one of the module’s slots, (2) Transferring the symbol contents of one slot to another, and (3) Requesting a DM recall. Modules in ACT-R all act concurrently, but the Procedural module sets up a central bottleneck in which only one production is allowed to fire at a time.

In ACT-R theory each production matching and execution cycle represents a single “step of cognition” (Anderson and Lebiere, 1998). Let’s look closer at the internal timing of this cycle^7^. First there is a “*matching phase*” where the if-clauses of all productions are matched against the current contents of all slots, checking if any productions have their list of constraints fully met. The Procedural module picks the satisfied production with the highest activation level (an analog parameter based on use history) and performs its actions. Next there is a “*pre-DM retrieval transfer phase*” in which the production directs the contents of some slots to be transferred to particular DM slots to prepare for a DM retrieval. Next there is a “*DM retrieval phase*” in which the empty slots of the DM module are filled based on recalling the memory which is the closest match. Finally there is a “*post-DM retrieval execution phase*” which may include additional slot transfers. This completes a single matching and execution cycle (i.e., a single “step of cognition”), the result being that some of the slot contents will have been changed. This may cause a different production to match in the next cycle and may cause external actions to be performed. Anderson (2007) has estimated that a single production cycle requires approximately 50 ms in the human brain (based on fitting model parameters to human timing performance data over a range of tasks).

A single machine language instruction run on a computer achieves very little on its own, but when many of these instructions are run in sequence as part of a program the result can be quite complex. The same analogy holds for ACT-R productions. A single production firing can achieve very little (for example, a single stimulus response), but the execution of dozens of these productions over a span of seconds has been used to model complex human thought processes and behaviors that occur at the same time scale.

ACT-R is widely used in the cognitive psychology community but has been mostly overlooked by the neuroscience community because its assumptions of symbol-containing slots, slot-to-slot transfers, and slot-to-slot equality comparisons have been seen as incompatible with biological brain circuits. The argument has been that ACT-R takes the “computer metaphor of the brain” too far. What I aim to show is that the symbol-containing slots, slot-to-slot transfers, and slot-to-slot equality comparisons assumed by ACT-R could be carried out in a biologically plausible manner if one assumes that all the ACT-R slots are implemented as separate partitions in a “global workspace” DPAAN.

#### Implementing basic ACT-R functionality using a DPAAN

A perceptual module could be implemented much like the perceptual DPAAN system depicted in Figure 2 – visual circuits (FH1 and FH2) would generate patterns that are unique for each perceptual attribute, and these patterns would be associated with global DPAAN stable states via the training of association networks like AN1, …, AN4. A motor module could be implemented much like the Vocalization unit in the DPAAN system depicted in Figure 2 – unique motor circuits would be driven by a DPAAN slot via an association network like AN5.

We have already seen how the Procedural module could be implemented – the Control Network depicted in Figure 7 is performing the role of an ACT-R style Procedural module. Recall that all productions in ACT-R are simply if-then rules where the if-clause is a list of constraints that may include a symbol being in a particular slot or an equality (or non-equality) of two slots. Such an if-clause is really just looking for a particular pattern of neural firing over a subset of the DPAAN’s slot neurons and its equality detection neurons. A production’s then-clause may specify that one of the DPAAN’s slots is overwritten with a new symbol or that the contents of one slot is transferred to another slot. Both of these operations can be initiated by flat activation vectors since symbol transfers can be initiated in the DPAAN by activating particular transfer control neurons. This means that the Procedural module’s entire store of production rules can be thought of as consisting of a single pattern associative network, one that associates a set of if-clause patterns with a set of then-clause patterns. Learning a new production would consist of training this pattern associative network in the Procedural module with a new pair of associations.

Figure 10 should assist in making this concept of a Procedural module as a single pattern associative network more concrete. In that figure the control network’s three “Rule” neurons (which we can now refer to as “production” neurons) are used to associate particular patterns of activity on the SD and Eq. neurons at the figure’s left side to generate particular output patterns on the transfer control neurons on the figure’s right side.

How could ACT-R’s DM module be implemented within the DPAAN framework? First a subset of the DPAAN’s slots would be specifically designated to be the interface slots of the DM module. Then a new set of synaptic connections would be created fully connecting all neurons within the DM module slots. This new set of synaptic connection (the DM store connections) imposes an additional set of stable attractor states on the set of neurons comprising the DM slots – these stable attractor states are the declarative memories of the system. Let’s say that three DPAAN slots (A, B, C) are designated to be the DM module slots. To retrieve a particular memory the Procedural module would first transfer symbols into slots A and B, and would force slot C into a blank state. Then the Procedural module would turn on the DM store’s synaptic connections which would drive all three slots toward the nearest stored DM stable attractor state. This would have the effect of filling in the contents of slot C based on the contents of A and B.

#### The count model

**Figure 12:**
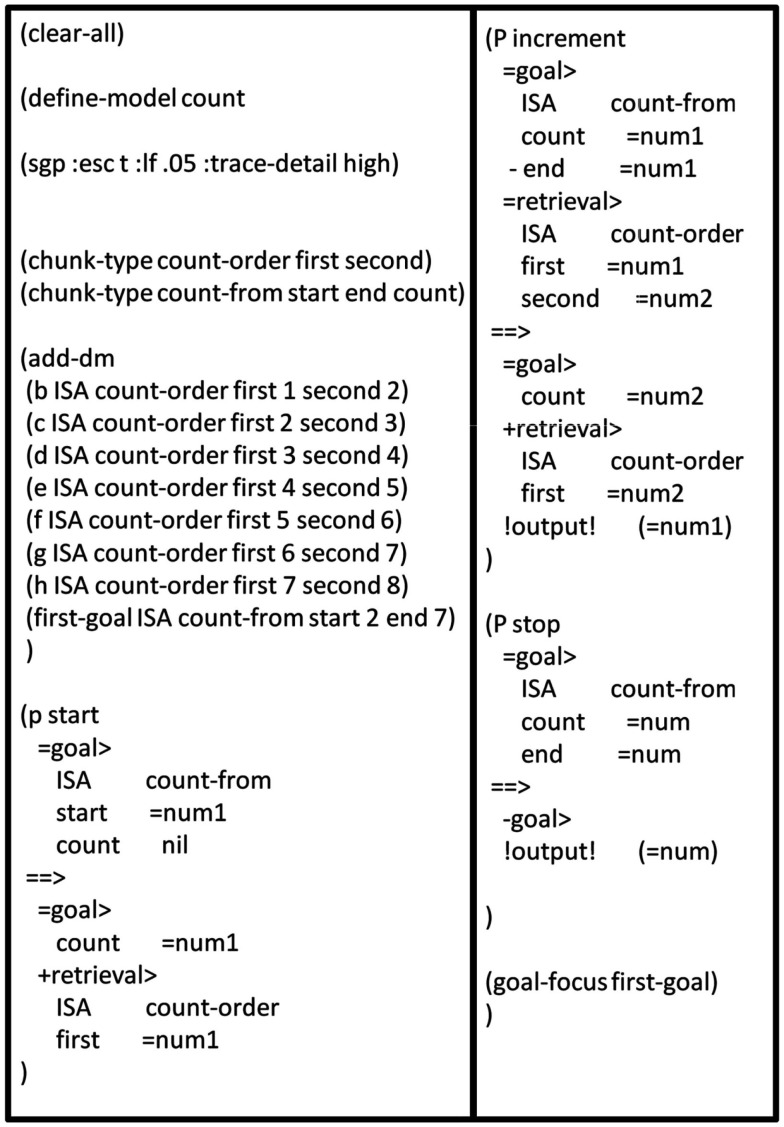
**Complete lisp definition file for the ACT-R count model**.

I will now demonstrate how one of the simplest ACT-R models, the Count Model, could be implemented using a DPAAN. The Count Model is an ACT-R model consisting of just three productions and a few DM chunks representing facts about the ordering of numbers. This model is described in the online ACT-R tutorial (http://act-r.psy.cmu.edu). Figure 12 shows the lisp definition file for the model.

The Count Model is a model of what goes on in a person’s mind when she counts from one number to another. First the model assumes a set of DM chunks specifying that “2” comes after “1,” that “3” comes after “2,” etc. The DM module is assumed to have three slots: “IsA,” “first,” and “second.” The chunk stating that “4” comes after “3” is defined in lisp as:

(d ISA count-order first 3 second 4)

This assigns the symbol “count-order” to the IsA slot, the symbol “3” to the first slot, and the symbol “4” to the second slot. The model also defines a chunk for the goal as follows:

(first-goal ISA count-from start 2 end 7)

This DM chunk is used to setup the initial conditions of the model. The model assumes that the Goal Module has four slots: “IsA,” “start,” “end,” and “count.” The “IsA” slot is assigned the symbol “count-from,” its “start” slot is assigned the symbol “2,” its “end” slot is assigned the symbol “7,” and its “count” slot is initialized to the symbol “nil.” This initialization of the Goal Module sets up the goal of counting from the number 2 to 7. If successful, during the course of running the model the symbol in the “count” slot should display the sequence “2”… “3”… “4”… “5”… “6”… “7.”

**Figure 13:**
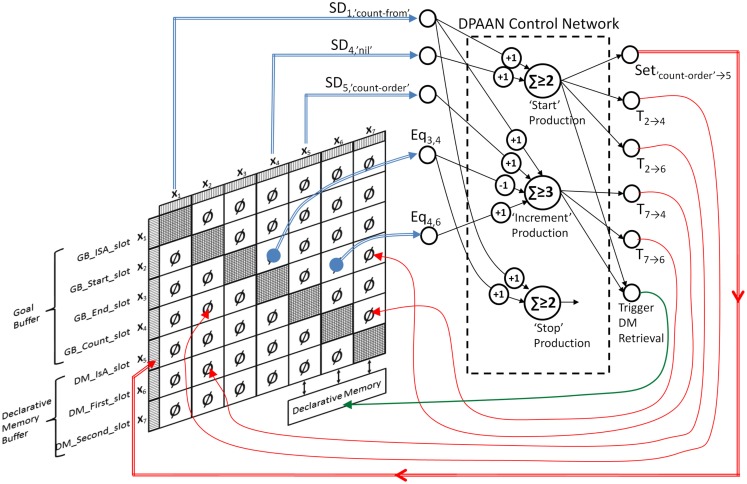
**Dynamically Partitionable AutoAssociative Network implementation of the ACT-R Count Model**.

There are seven slots in total, four in the Goal Module (“IsA,” “start,” “end,” “count”), and three in the DM module (“IsA,” “first,” “second”). The 11 symbols posited by the model are: “one,” “two,” “three,” “four,” “five,” “six,” “seven,” “eight,” “count-from,” “count-order,” and “nil.” In order to implement this model in neural network form using a DPAAN we first create a DPAAN having seven slots as depicted in Figure 13.

DPAAN slots **X_1_**, **X_2_**, **X_3_**, **X_4_** correspond to the Goal Module slots, and **X_5_**, **X_6_**, **X_7_** correspond to the DM Module slots. These are shown with double arrowed connections to a box labeled Declarative Memory. To model the symbols of the ACT-R model, the DPAAN’s synaptic weights are trained to have 11 stable attractor states, 1 corresponding to each of the 11 symbols in the model.

Now let’s look at the three productions defined in the Count Model, and convert each to a rule neuron like in Figure 10. The lisp syntax for these productions may be obscure to those unfamiliar with ACT-R modeling so I will describe each in words:

The “start” production is designed to fire at the start of the counting operation, setting up the modules’ contents for the task. It is implemented by the “start” production neuron as shown in Figure 13. Its if-clause checks that the Goal module’s IsA slot contains the symbol “count-from.” This is implement by a synapse of weight +1 tying the neuron to a symbol detection neuron SD_1,“count-from”_. The if-clause also checks that the “count” slot contains the symbol “nil.” This is implemented by a synapse of weight +1 to SD_4,“nil”_. The threshold of the neuron is set to ≥ 2 which means that the neuron will fire only if both of these conditions are met. The then-clause of the “start” production directs several actions: (1) Setting of the DM module’s IsA slot to the symbol “count-order,” (2) Transfer of the contents of the “start” slot to the “count” slot, (3) Transfer of the contents of the “start” slot to the DM module’s “first” slot, and (4.) Triggering a DM retrieval. In our DPAAN implementation these actions are triggered by the neuron’s output being tied to the neurons Set_“count-order”→5_, T_2 → 4_, T_2 → 6_, and the DM trigger neuron.

The “increment” production is designed to do the actual counting. It is implemented by the “increment” production neuron. The if-clause checks that the Goal module’s IsA slot contains the symbol “count-from.” This is implement by a +1 synapse tying the neuron to SD_1,“count-from”_. It also checks that the DM module’s “IsA” slot contains the symbol “count-order” (checking that a DM fact about count-order is present). This is implemented by a +1 synapse from SD_5,“count-order”_. It also checks that the contents of the Goal module’s “end” and “count” slots are not equal. This is done to allow the model to stop counting when it has reached the target number. This is implemented by a −1 synapse from the Eq_3,4_ neuron. The negative weight allows this equality detection neuron to veto the action of the “increment” production. Finally it checks if the “count” slot is equal to the DM module’s “first” slot. This is to check if the appropriate count-order fact is loaded. This is implemented by a +1 synapse from Eq_4,6_. The threshold of the neuron is set to ≥3. The then-clause of the “increment” production directs several actions: (1) Transfer of the contents of the DM module’s “second” slot to the Goal module’s “count” slot, (2) Transfer of the of the contents of the “second” slot to the “first” slot, and (3) The triggering of a DM retrieval. These actions are triggered by the neuron’s output being tied to T_7 → 4_, T_7 → 6_, and the DM trigger neuron.

The “stop” production is designed to fire when the Goal module’s “count” slot reaches the target value held in the “end” slot. The “stop” production is implemented by the “stop” production neuron, receiving a +1 synapses from SD_1,“count-from”_ and Eq_3,4_. In the lisp model, the production causes the ACT-R execution to stop. In our model the output of the “stop” production neuron is left unwired.

#### Simulation results

A simulation was written for the network in Figure 13. Matlab simulation files are provided in the supplementary online material.

The program first defines a DPAAN comprised of seven slots containing 100 neurons each, corresponding to the seven slots in Figure 13. As in prior simulations, a symbol vocabulary is created for the DPAAN by creating a set of random neural activation vectors, one for each of the model’s symbols. These are used to generate the weight matrix of the DPANN.

Next DM vectors are created corresponding to each of the seven “count-order” chunks. Consider the DM chunk:

(d ISA count-order first 3 second 4)

If the DM module held this chunk then the pattern of activity over slots **X_5_**, **X_6_**, **X_7_** would be:

$$\left[{\text{X}}_{5};{\text{X}}_{6};{\text{X}}_{7}\right]=\left[{\text{P}}_{5}^{\prime \text{count}-\text{order}\prime };{\text{P}}_{6}^{\prime \text{three}\prime };{\text{P}}_{\text{7}}^{\prime \text{four}\prime }\right]$$

This vector must be stored in the DM. Here are all of the memory vectors to store:

$$\begin{array}{rcll} \left[{\text{X}}_{5};{\text{X}}_{6};{\text{X}}_{7}\right] & =\left[{\text{P}}_{5}^{\prime \text{count}-\text{order}\prime };{\text{P}}_{6}^{\prime \text{one}\prime };{\text{P}}_{\text{7}}^{\prime \text{two}\prime }\right] & & \\ \left[{\text{X}}_{5};{\text{X}}_{6};{\text{X}}_{7}\right] & =\left[{\text{P}}_{5}^{\prime \text{count}-\text{order}\prime };{\text{P}}_{6}^{\prime \text{two}\prime };{\text{P}}_{\text{7}}^{\prime \text{three}\prime }\right] & & \\ \left[{\text{X}}_{5};{\text{X}}_{6};{\text{X}}_{7}\right] & =\left[{\text{P}}_{5}^{\prime \text{count}-\text{order}\prime };{\text{P}}_{6}^{\prime \text{three}\prime };{\text{P}}_{\text{7}}^{\prime \text{four}\prime }\right] & & \\ \left[{\text{X}}_{5};{\text{X}}_{6};{\text{X}}_{7}\right] & =\left[{\text{P}}_{5}^{\prime \text{count}-\text{order}\prime };{\text{P}}_{6}^{\prime \text{four}\prime };{\text{P}}_{\text{7}}^{\prime \text{five}\prime }\right] & & \\ \left[{\text{X}}_{5};{\text{X}}_{6};{\text{X}}_{7}\right] & =\left[{\text{P}}_{5}^{\prime \text{count}-\text{order}\prime };{\text{P}}_{6}^{\prime \text{five}\prime };{\text{P}}_{\text{7}}^{\prime \text{six}\prime }\right] & & \\ \left[{\text{X}}_{5};{\text{X}}_{6};{\text{X}}_{7}\right] & =\left[{\text{P}}_{5}^{\prime \text{count}-\text{order}\prime };{\text{P}}_{6}^{\prime \text{six}\prime };{\text{P}}_{\text{7}}^{\prime \text{seven}\prime }\right] & & \\ \left[{\text{X}}_{5};\;{\text{X}}_{6};\;{\text{X}}_{7}\right] & =\left[{\text{P}}_{5}^{\prime \text{count}-\text{order}\prime };{\text{P}}_{6}^{\prime \text{seven}\prime };{\text{P}}_{\text{7}}^{\prime \text{eight}\prime }\right] & & \end{array}$$

As described above, these memories could potentially be stored as an additional set of synaptic weights among the **X_5_**, **X_6_**, **X_7_** neurons. In the software simulation this DM store was instead simply implemented by a look-up table.

**Figure 14:**
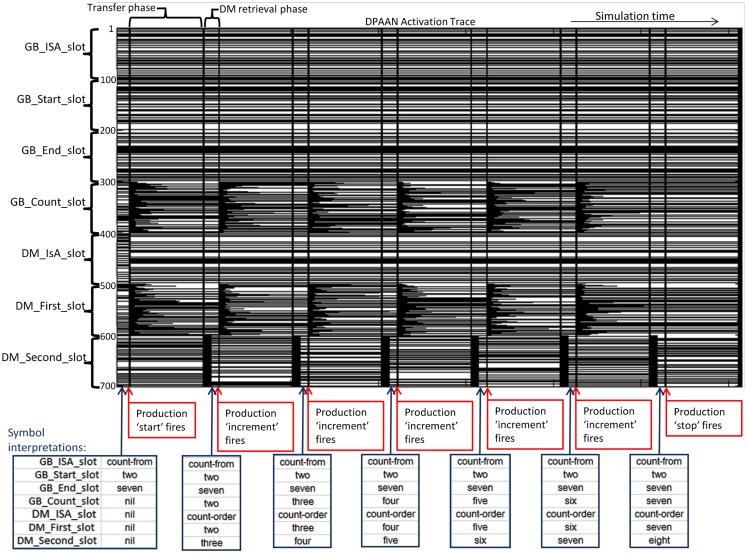
**Simulation results of the DPAAN implementation of the ACT-R count model**. (White = active neuron, black = inactive neuron).

Figure 14 shows the results of the simulation by plotting the activity of all 700 DPAAN neurons over the course of the simulation. Below the neural activity trace are symbolic interpretations of the contents of the DPAAN slots at particular points in the simulation as well as labels showing when particular production neurons fired.

The DPAAN neurons are initialized as described above: setting the Goal module’s “IsA,” “start,” and “end” slots to “count-from,” “two,” and “seven” respectively, and setting all other slots to “nil.” After 10 simulation steps the production neurons’ activities are evaluated. The “start” production neuron is found to have exceeded threshold and thus directs a setting of slot **X_5_**, and the transfers from slot **X_2_** to **X_4_**, and from **X_2_** to **X_6_**. The simulation gives this transfer 40 simulated time steps to complete. Then the DM retrieval is carried out by first setting slot **X_7_** to blank (no activation) for five time steps and then performing a DM retrieval based on the stored memory vectors listed above. After another five time steps the production neurons’ activities are revaluated thus starting the match-execute cycle over again. This match-execute cycle is repeated a total of seven times during the simulation.

Looking at the symbolic interpretations displayed below the neural activity trace one can see that first the “start” production fires, then the “increment” production fires five times in a row, and finally the “stop” production fires. Looking at the Goal module’s “count” slot one can see that it goes through the symbol sequence: “two” … “three” … “four” … “five” … “six” … “seven” over the course of the simulation. This is precisely the behavior that the ACT-R Count Model displays when its lisp code is run in the traditional fashion.

## Summary and Conclusion

The concept of *assigning* a *symbol* to a *variable* is central to computer science. Ever since the foundational work of Alan Turing, John von Neumann, and others, the computer science community has had a clear understanding of how such algorithmic-level concepts can be mapped onto physical computer hardware. We know that in a computer a *symbol* is physically instantiated by a particular pattern of high and low voltage levels that can be stored in any of the computer’s memory registers. A *variable* is physically instantiated as a memory register; and to *assign* a particular *symbol* to a *variable* one sets the bits of the register to match the symbol’s unique binary pattern. At the electrical level, this setting of bit values is accomplished by *transferring* the voltages of the source register’s bits to the target register’s bits in a one-to-one fashion via a wire bus and a set of tri-state buffers.

The concept of *assigning* a *symbol* to a *variable* is also central to cognitive science’s understanding of how the mind works (as exemplified in the ACT-R theory). Unfortunately our neuroscience understanding of how concepts like *symbol*, *variable*, and *assignment* are mapped onto the neural circuits has been much less precise. This is why there is a binding “problem” in neuroscience but not in computer science. The DPAAN theory outlined above is designed to offer a precise hypothesis for how these concepts are mapped (in a biologically plausible manner) onto the brain’s neural circuitry.

In the DPAAN theory every symbol *S*^1^, *S*^2^, …, *S^L^* that the brain can token is associated with a unique stable attractor state (designated ***P***^1^, ***P***^2^, …, ***P****^L^*) in a global workspace of neurons. This global workspace (the DPAAN network) can be dynamically partitioned into separate subnetworks (partitions) by temporarily setting to zero all synapses connecting neurons in separate partitions. The resulting subnetworks each retain all of the stable attractor states that were written into the full DPAAN’s synaptic matrix in the sense that the *k*th partition will inherit stable attractor states ${\text{P}}_{k}^{1},\;{\text{P}}_{k}^{2},\;\ldots ,\;{\text{P}}_{k}^{L}$ – each one a *piece* of one of the DPAAN’s attractor states.

In the DPAAN theory, a *variable* is physical instantiated as a particular partition of the global DPAAN. Therefore a DPAAN with *K* partitions (**x**_1_, **x**_2_, …, **x***_k_*) is seen as implementing *K* variables (*X*_1_, *X*_2_, …, *X_K_*). We say that *variable* *X_k_* contains *symbol* *S^l^* if the neural activation pattern in the *k*th partition is ${\text{x}}_{k}={\text{P}}_{k}^{1}.$ The neural mechanics of assigning the symbol in variable *X_k_* to variable *X_k_’* simply consists of un-zeroing the synapses from the *k* partition to the *k*′ partition, thus allowing the stable attractor dynamics of the network to drive the *k*′ partition into part of the same global attractor state that the *k* partition is currently in.

This is the key feature of the DPAAN formulation that makes it biologically plausible – a symbol is not associated with a particular pattern of activation (as it would be in a human-designed computer); instead, a DPAAN symbol is associated with a *piece* of one of the full DPAAN’s attractor states. It is biologically plausible to assume that the brain contains a set of interconnected cortical areas which together act as a global associative memory; and it is biologically plausible to assume that the brain has control circuitry which can selectively suppress (and unsuppress) particular blocks of synapses projecting between different cortical regions. If the biological brain implements these features then it can (by using this DPAAN method) implement *symbol assignments* to *variables* while avoiding any requirement for the type of one-to-one wiring seen in a human-designed computer.

In the three models and simulations above I demonstrated how the DPAAN formulation could be used to solve the neural binding problem. The first was a simple perceptual model (Figure 2) which demonstrated how association networks can be trained to “ground” DPAAN symbols, associating these symbols with particular features of the external world. This perceptual model also demonstrated the basics of how a symbol in one DPAAN partition can be assigned to another. For example, Figure 5 showed how the symbol “CIRCLE” can be transferred from slot #2 to slot #5 of the DPAAN, thus showing how the perceptual associations learned on one set of neurons (slot #2) and the motor associations learned on another set of neurons (slot #5) could both be utilized with the same symbol (“CIRCLE”). This is a direct example of how a DPAAN can provide “role-filler independence” (Hummel et al., 2004; Hummel, 2011).

The second model (Figure 8) demonstrated how syntax-sensitive rules could be implemented using a DPAAN. Key to this was a set of equality detection neurons, each sensitive to the Hopfield energy function calculated over the set of synapses connecting the two slots being compared. These equality detection neurons are essentially asking the question: “Are the activation patterns in these two slots actually different pieces of the same global attractor state?” By framing the equality detection problem in this way, syntax-sensitive rule neurons (like those shown in Figure 10) can be implemented simply as pattern recognition neurons whose outputs drive particular transfer control neurons controlling blocks of synapses in the DPAAN.

The third model (Figure 13) demonstrated how a DPAAN (augmented with rule neurons and a DM module) can implement the core symbol processing pieces of the ACT-R production system. Although the DPAAN’s ability to accomplish this was demonstrated with one of the simplest ACT-R model (the Count Model), this same approach could be used to implement much more complex ACT-R models composed of hundreds of production rules and DM chunks.

This neural implementation of ACT-R’s symbol processing core also suggests ways in which ACT-R’s analog and symbolic learning mechanisms could be neurally implemented. For example, learning a new production in an ACT-R model would consist of training the DPAAN rule network with a new associative pattern linking the symbol detection and equality detection neurons with the transfer control neurons. The ACT-R theory includes detailed models of “symbolic” production learning (based on a mechanism called production compilation), “analog” production learning (based on adapting the relative likelihood of production firing based on use history), and “symbolic” and “analog” DM learning (Anderson, 2007). These more complex aspects of the ACT-R architecture are well beyond the scope of this paper, but the DPAAN implementation of ACT-R’s symbol processing core described here readily suggests ways in which some of these more complex aspects of ACT-R theory might be neurally implemented.

In recent years, researchers have begun to map particular ACT-R modules onto specific cortical and subcortical brain regions (see Anderson, 2007; Anderson et al., 2007). For example, the fusiform gyrus is associated with ACT-R’s Visual buffer, and a region of the perfrontal cortex is associated with the DM module’s retrieval buffer. ACT-R’s all-important Procedural module has been associated with the basal ganglia implying that neural circuits within the basal ganglia are somehow routing symbolic information between these other cortical areas (discussed in Stocco et al., 2008). Although there is a wealth of indirect evidence that the basal ganglia may be serving such a role, there has never been a clear understanding of what such “routing” actually entails at the neural level. The DPAAN theory presented here represents a clear hypothesis as to what such “routing” actually entails. The DPAAN theory predicts that long distance connections among these cortical areas functionally combine all of these cortical regions into a single global autoassociative network containing multiple stable attractor states (i.e., this network of cortical buffer regions is the brain’s global DPAAN). The role of the basal ganglia then is to selectively turn on or off some of these long distance projections so as to dynamically partition this global cortical network – this is what “routing” actually entails according to the DPAAN theory.

In summary, the DPAAN theory provides a solution to the neural binding problem requiring only “flat” neural activation vectors. As such it is directly compatible with the most well developed neural models of learning, memory, and pattern recognition. The DPAAN theory appears biologically plausible given that it calls for very few additional assumptions beyond these established neural models – the main additional assumptions being that the brain contains neurons which can selectively turn on or off blocks of synaptic connections (the transfer control neurons), and that the brain contains neurons which approximate calculating the Hopfield energy function over a set of synapses (the equality detection neurons). These assumptions about brain wiring can be taken as predictions of the DPAAN theory. Most significantly, if the DPAAN theory proves correct then it would offer an explanation for how our most successful model of the mind (the ACT-R theory) is physically implemented in the hardware of the brain.

## Conflict of Interest Statement

The author declares that the research was conducted in the absence of any commercial or financial relationships that could be construed as a potential conflict of interest.

## Footnotes

^1^The term “conjunctive coding” (Hummel et al., 2004) is sometimes used to describe a neural representation which uses one set of neurons to represent the color of object #1 and a separate set to represent the color of object #2. As such I could refer to this as the “conjunctive coding hypothesis.” I avoid doing this however since the concept of conjunctive coding has gained a number of additional assumptions over the years for how such a neural representation is interpreted by the larger neural system, assumptions which I will not be making here. Therefore I am instead using a more generic nomenclature “anatomical binding” which simply implies that different sets of neurons are used, in some way, to represent the same symbol (e.g., “red”) in different contexts.

^2^It is important to understand that the DPAAN solution presented here does *not* depend on the truth of this multiple spotlights hypothesis. This multiple spotlights extension of the Standard Model of the visual system is merely being used here as a concrete and straightforward example of how the anatomical binding hypothesis enters into a model capable of representing multiple objects simultaneously. The DPAAN theory can actually answer some of the main arguments made against such a multiple spotlight model by showing how the binding issues that it raises can be solved. Hayworth (2009) and Hayworth et al. (2011) present experimental and simulation results supporting a Multiple Slots Multiple Spotlights model of the visual system and discuss how the classic binding problem arises in such a model.

^3^The figure depicts FH1 and FH2 as being completely independent (i.e., as not sharing any cells except for the sharing of the same retinal input). This is unlikely to be the case in the real brain, but this is not important to the present discussion. What is an important assumption of this model is the independence of the two spotlights of attention and the independence of the final sets of neurons (**X_1_**; **Y_1_** and **X_2_**; **Y_2_**) which encode the properties of the objects they highlight.

^4^It is important to understand that I am using human languages here simply as an analogy for the different neural codes used by **X_1_**, **X_2_**, and **voc** to communicate with each other inside the brain. The motor field **voc** can also be thought of as understanding the language it uses to vocalize to the external world but this fact has nothing to do with the analogy presented here.

^5^A reviewer has pointed out that the connections within this central DPAAN in effect contain the multiple “language savants” described earlier. However, unlike the separate language savants, the DPAAN, by being globally pre-trained as an autoassociative memory, ensures that all translations are perfectly synchronized thereby ensuring role-filler independence across all slots of the DPAAN. A connected module thus needs only train its local association network to the DPAAN’s one universal language. These concepts will become clearer when demonstrated in the example perceptual network of the section entitled “Example #1: Use of a DPAAN to Solve the Perceptual Binding Problem.”

^6^Note that this energy function is written in a form which assumes that neural activations take on values of +1 and −1 as opposed to the +1 and 0 values described in the text above. This is to match what was done in the simulations described in later sections.

^7^The description here is somewhat simplified relative to the actual ACT-R model, and the nomenclature used for describing the phases has been altered to better match the simulation below.
